# Thio-Schiff bases derived from 2,2′-disulfanedianiline via nanocerium oxide: antimicrobial effect and antiproliferative effects in melanoma cells

**DOI:** 10.55730/1300-0527.3414

**Published:** 2022-03-02

**Authors:** Aslıhan DALMAZ, Sefa DURMUŞ, Görkem DÜLGER, Merve ALPAY

**Affiliations:** 1 Department of Natural and Herbal Products/Cosmetic Product, Health Science Institute, Düzce University, Düzce, Turkey; 2 Department of Chemistry, Faculty of Arts and Science, Düzce University, Düzce, Turkey; 3 Department of Medical Biology, Faculty of Medical, Düzce University, Düzce, Turkey; 4 Department of Medical Biochemistry, Faculty of Medical, Düzce University, Düzce, Turkey

**Keywords:** Schiff base, nanocatalyst CeO_2_, antimicrobial activity, cytotoxicity, antiproliferative, melanoma

## Abstract

In this study, the synthesis of dimeric disulfide-Schiff bases was carried out using two methods. The structures of the obtained Schiff bases were elucidated by various spectroscopic methods as well as elemental analysis. The Schiff base derivative compounds **(3a-6)** were screened for in vitro antibacterial activity against multidrug-resistant microorganisms using microdilution method. All the tested compounds showed varying inhibition zones against the pathogens. According to MIC results, the compound 2 was shown strong inhibitory activity against all the tested microorganisms compared to antibiotics. In addition, all the tested compounds showed different antiproliferative effects on the melanoma cell line (B16F10). Our synthesized dimeric disulfide-Schiff bases have shown significant various effects.

## 1. Introduction

Cerium dioxide (CeO_2_), or ceria, is a crystalline material which is a very significant compound belonging to the beneficial rare-earth family. Ceria has received more attention of late due to many distinctive features, such as its unique ultraviolet radiation-absorbing capability [1,2] as well as its high thermal stability, high hardness and reactivity [3]. Nanoceria exhibits an extremely high performance in many applications including as material for catalysts (CO oxidation) and UV inhibitors. It is also effectively used in chemical-mechanical polishing, in fuel cells and for water treatment [4–7].

Schiff bases and their structural derivatives, as binding agents containing acyclic and cyclic imine C=N bonds, are highly significant in contemporary coordination chemistry [8]. Disulfides are also frequently used as reagents in organic syntheses as a substitute for many anions and as a protective group for thiols [9,10]. In addition, disulfides are generally preferred in the vulcanization of rubber and elastomer [11]. The most striking feature of many Schiff-base compounds is that they can be easily synthesized using relatively inexpensive materials. Schiff-base compounds have many applications due to the presence of the -C=N group, electronegative nitrogen, the sulfur and oxygen atoms in the molecule. For instance, Schiff bases and dimeric disulfide-Schiff bases derivatives are particularly substantial compounds in medicinal chemistry. The therapeutic effects of Schiff bases such as antimicrobial and anticarcinogenic activity are known. As they are superior reagents, Schiff bases are frequently chosen for biological, pharmacological, clinical and analytical applications [12,13].

The rapid emergence of multidrug-resistant organisms poses a crucial threat to global health. Drug-resistant pathogens are a growing menace to the entire population. Over the past 20 years, many researchers have tried to develop new and effective antimicrobial reagents that are both resistant-free and cost effective. Sulfur is one of the fundamental elements necessary for life. Schiff bases-bearing disulfide bonds are known to exhibit biological activity [14], including anti-HIV [15], anticancer [16], antifertility and antioxidant properties.

Malignant melanoma is the most aggressive form of skin cancer and is the cause of 80% of all skin cancer deaths. This skin tumor is caused by the melanocytes which give color to the skin. In men, melanoma is commonly seen on the shoulders and hips or on the head and neck, while in women it is more often found on the arms and legs. Although it can be successfully treated in its early stages, melanoma can reoccur at any time during the first 10 years after treatment. In this case, standard cancer treatment methods such as surgery, radiotherapy and chemotherapy may not be successful [17]. Metallodrugs are used as long-acting antitumor medicines. These types of molecules are particularly interesting as a potential source of new antimelanoma drugs because of their redox-active characteristics, considering that melanoma exhibits high amounts of reactive oxygen species [18,19].

In recent years, because of their complexity and anticancer activity, interest in Schiff bases has become increasingly prevalent in the medical world and they are being investigated as reactive agents in the fight against cancer [20].

When the literature is examined, we found that there are few studies on the melanoma cell line of thio-Schiff bases derived from 2,2′-disulfanedilaniline. Since we thought that there would be a supporting literature in this direction, we investigated the synthesis, antimicrobial effect, especially cytotoxic and antiproliferative effect of dimeric disulfide-Schiff bases derived from 2,2′-disulfanedylaniline.

In this study, the synthesis of dimeric disulfide-Schiff bases was carried out with and without using a nanocatalyst. Schiff bases derived from 2,2′-disulfanediyldianiline were characterized by using Fourier transform infrared spectroscopy (FTIR), nuclear magnetic resonance (NMR) spectroscopy, mass spectroscopy (MS) and elemental analysis (EA).

## 2. Materials and methods

### 2.1. Chemicals and instrumentation

All reagents were supplied commercially. The solvents and the compounds of 2-aminothiophenol, 2-hydroxy benzaldehyde, 2-hydroxy-5-bromo-benzaldehyde, 2-hydroxy-3-methoxy benzaldehyde and sodium hydroxide were purchased from Merck, the cerium(III) nitrate hexahydrate and 4-hydroxy-3-nitro benzaldehyde from Acros, the 2-hydroxy-3-nitro benzaldehyde and 2-hydroxy-5-nitro benzaldehyde from Sigma Aldrich and the 3-methyl salicylaldehyde and 5-methyl salicylaldehyde from TCI.

The morphology and crystal properties of the synthesized CeO_2_ nanocatalyst were determined. Compound structures were investigated using Fourier transform infrared spectroscopy (FTIR), nuclear magnetic resonance (NMR) spectroscopy and mass spectroscopy (MS). The Fourier transform infrared-attenuated total reflection spectroscopy (FTIR-ATR) results were recorded via a Perkin Elmer spectrometer and the wave numbers were averaged across the spectral range of 550–4000 cm^−1^. The ^1^H NMR spectra were recorded in CDCl_3_ on a Bruker spectrometer at 400 MHz. The ^13^C NMR spectra were recorded in CDCl_3_ and DMSO-*d_6_* on a Bruker spectrometer operating at 100 MHz. All chemical shifts were reported in *δ* (ppm) using tetramethylsilane (TMS) as an internal standard. The elemental analyses (EA) were carried out using the Thermo Scientific Flash 2000 analyzer. Mass spectra were obtained with an AB SCIEX 4000 Q-TRAP LC-MS/MS instrument. Melting points were determined using a Stuart Equipment apparatus.

### 2.2. Synthesis of compounds

The CeO_2_ nanocatalyst was prepared in accordance with the method described in previous studies [21–23]. The 2,2′-diaminodiphenyl disulfide was synthesized via the oxidation of 2-aminothiophenol. The compounds were synthesized both with and without using the CeO_2_ nanocatalyst, as described in previous studies [21].

#### 2.2.1. 2,2′-disulfanediyldianiline (2)

Shiny yellow, yield 75%, mp: 90–92 °C. FTIR *ν*_max_ 3375, 1471, 744 cm^−1^. ^1^H NMR (400 MHz, CDCl_3_): *δ* 4.31 (s, 2H, NH_2_), 6.56 (td, *J* = 7.5, 1.2 Hz, 1H, Ar-H), 6.66 (dd, *J* = 8.5, 1.3 Hz, 1H, Ar-H), 7.19–7.07 (m, 2H, Ar-H). ^13^C NMR (101 MHz, CDCl_3_): *δ* 148.7, 136.9, 131.7, 118.8, 118.2, 115.3. MS *m/z* 249.3 [*M*+H]^+^. Anal. calcd for C_12_H_12_N_2_S_2_: C, 58.03; H, 4.87; N, 11.28; S, 25.8; found: C, 58.01; H, 4.69; N, 11.14; S, 26.16.

#### 2.2.2. 6,6′-((1Z,1’Z)-((disulfanediylbis(2,1-phenylene))bis(azaneylylidene))bis (methaneylylidene))bis(2-methylphenol) (3a)

Shiny yellow; yield 90%, mp: 185 °C. FTIR *ν*_max_ 2906, 1610, 1459, 1254, 738, 563 cm^−1^. ^1^H NMR (400 MHz, CDCl_3_): *δ* 2.33 (s, 3H, CH_3_), 6.86 (t, *J =* 7.5 Hz, 1H, Ar-H), 7.14 (dd, *J =* 7.6, 1.4 Hz, 1H, Ar-H), 7.18 (dd, *J* = 7.6, 1.6 Hz, 1H, Ar-H), 7.21 (dd, *J* = 7.5, 1.5 Hz, 1H, Ar-H), 7.25 (dd, *J* = 9.0, 7.6 Hz, 2H, Ar-H), 7.65 (dd, *J* = 7.7, 1.4 Hz, 1H, Ar-H), 8.58 (s, 1H, H-C=N), 13.00 (s, 1H, O-H). ^13^C NMR (101 MHz, CDCl_3_): *δ* 163.03, 159.49, 134.72, 131.62, 130.35, 127.63, 127.53, 127.01, 126.95, 126.52, 118.77, 118.44, 117.61, 15.66. MS *m/z* 485.7 [*M*+H]^+^. Anal. calcd for C_28_H_24_N_2_O_2_S_2_: C, 69.39; H, 4.99; N, 5.78; S, 13.23; found: C, 69.46; H, 4.95; N, 5.75; S, 13.15.

#### 2.2.3. 2,2′-((1E,1’E)-((disulfanediylbis(2,1-phenylene))bis (azaneylylidene))bis(methaneylylidene))bis(4-methylphenol) (3b)

Pale yellow; yield 93%, mp: 190 °C. FTIR *ν*_max_ 2911, 1610, 1428, 1277, 749, 570 cm^−1^. ^1^H NMR (400 MHz, CDCl_3_): *δ* 2.33 (s, 3H, CH_3_), 6.96 (d, *J* = 8.1 Hz, 1H, Ar-H), 7.13 (dd, *J* = 7.6, 1.2 Hz, 1H, Ar-H), 7.17 (dd, *J* = 7.4, 1.4 Hz, 1H, Ar-H), 7.21 (d, *J* = 9.0 Hz, 2H, Ar-H), 7.26–7.23 (m, 1H, Ar-H), 7.65 (dd, *J* = 7.7, 1.3 Hz, 1H, Ar-H), 8.57 (s, 1H, H-C=N), 12.64 (s, 1H, O-H). ^13^C NMR (101 MHz, CDCl_3_): *δ* 162.85, 134.66, 132.54, 131.62, 128.32, 127.67, 127.65, 127.57, 127.12, 127.07, 118.83, 117.60, 117.25, 20.37. MS *m/z* 485.7 [*M*+H]^+^. Anal. calcd for C_28_H_24_N_2_O_2_S_2_: C, 69.39; H, 4.99; N, 5.78; S, 13.23; found: C, 69.30; H, 5.04; N, 5.82; S, 13.29.

#### 2.2.4. 6,6′-((1E,1’E)-((disulfanediylbis(2,1-phenylene))bis(azaneylylidene))bis (methaneylylidene))bis(2-nitrophenol) (4a)

Orange; yield 100%, mp: 228 °C. FTIR *ν*_max_ 1619, 1458, 1345, 1281, 741, 595 cm^−1^. ^1^H NMR (400 MHz, CDCl_3_): *δ* 7.16 (dd, *J* = 8.9, 0.6 Hz, 2H, Ar-H), 7.24 (d, *J* = 8.1 Hz, 2H, Ar-H), 7.73–7.69 (m, 1H, Ar-H), 8.33 (dd, *J* = 9.4, 2.9 Hz, 1H, Ar-H), 8.44 (d, *J* = 2.6 Hz, 1H, Ar-H), 8.66 (s, 1H, H-C=N), 13.85 (s, 1H, O-H). ^13^C NMR (101 MHz, DMSO-*d_6_*): *δ* 191.61, 163.99, 155.42, 145.43, 138.87, 138.27, 130.96, 129.81, 129.48, 129.24, 128.73, 121.85, 119.49. MS *m/z* 547.5 [*M*+H]^+^. Anal. calcd for C_26_H_18_N_4_O_6_S_2_: C, 57.14; H, 3.32; N, 10.25; S, 11.73; found: C, 57.07; H, 3.42; N, 10.32; O, S, 11.67.

#### 2.2.5. 2,2′-((1E,1’E)-((disulfanediylbis(2,1-phenylene))bis(azaneylylidene)) bis(methaneylylidene))bis(4-nitrophenol) (4b)

Orange; yield 97%, mp: 234 °C. FTIR *ν*_max_ 1613, 1472, 1337, 1292, 746, 575 cm^−1^. ^1^H NMR (400 MHz, CDCl_3_): *δ* 7.14 (dd, *J* = 8.9, 0.6 Hz, 2H, Ar-H), 7.20 (d, *J* = 8.1 Hz, 2H, Ar-H), 7.71–7.68 (m, 1H, Ar-H), 8.31 (dd, *J* = 9.4, 2.9 Hz, 1H, Ar-H), 8.42 (d, *J* = 2.6 Hz, 1H, Ar-H), 8.70 (s, 1H, H-C=N), 13.83 (s, 1H, O-H). ^13^C NMR (101 MHz, DMSO-d_6_): *δ* 189.50, 166.20, 158.20, 140.17, 138.56, 138.00, 131.04, 128.62, 128.34, 127.5, 124.83, 122.64, 118.93. MS *m/z* 547.5 [*M*+H]^+^. Anal. calcd for C_26_H_18_N_4_O_6_S_2_: C, 57.14; H, 3.32; N, 10.25; S, 11.73; found: C, 57.19; H, 3.26; N, 10.35; S, 11.67.

#### 2.2.6. 4,4′-((1E,1’E)-((disulfanediylbis(2,1-phenylene))bis(azaneylylidene))bis (methaneylylidene))bis(2-nitrophenol) (4c)

Bright orange; yield 95%, mp: 210 °C. FTIR *ν*_max_ 3196, 1620, 1533, 1487, 1279, 754, 565 cm^−1^. ^1^H NMR (400 MHz, CDCl_3_): *δ* 7.06 (dd, *J* = 7.2, 1.9 Hz, 1H, Ar-H), 7.24–7.15 (m, 2H, Ar-H), 7.30 (d, *J* = 8.8 Hz, 1H, Ar-H), 7.66 (dd, *J* = 7.6, 1.6 Hz, 1H, Ar-H), 8.38 (dd, *J* = 8.8, 2.0 Hz, 1H, Ar-H), 8.46 (s, 1H, Ar-H), 8.56 (d, *J* = 2.0 Hz, 1H, H-C=N), 10.90 (s, 1H, O-H). ^13^C NMR (101 MHz, DMSO-*d_6_*): *δ* 159.15, 155.42, 148.40, 137.40, 134.75, 131.50, 127.89, 127.78, 127.67, 127.14, 125.60, 120.31, 118.37. MS *m/z* 547.5 [*M*+H]^+^. Anal. calcd for C_26_H_18_N_4_O_6_S_2_: C, 57.14; H, 3.32; N, 10.25; S, 11.73; found: C, 57.20; H, 3.28; N, 10.22; S, 11.80.

#### 2.2.7. 2,2′-((1E,1’E)-((disulfanediylbis(2,1-phenylene))bis(azaneylylidene))bis (methaneylylidene))bis(4-bromophenol) (5)

Yellow; yield 95%, mp: 182–185 °C. FTIR *ν*_max_ 1609, 1460, 1276, 735, 624, 557 cm^−1^. ^1^H NMR (400 MHz, CDCl_3_): *δ* 6.96 (d, *J* = 8.8 Hz, 1H, Ar-H), 7.15–7.12 (m, 1H, Ar-H), 7.25–7.21 (m, 1H, Ar-H), 7.29 (d, *J* = 1.4 Hz, 1H, Ar-H), 7.55–7.46 (m, 2H, Ar-H), 7.66 (dd, *J* = 7.7, 1.4 Hz, 1H, Ar-H), 8.53 (s, 1H, H-C=N), 12.85 (s, 1H, O-H). ^13^C NMR (101 MHz, CDCl_3_): *δ* 161.43, 160.17, 146.22, 136.22, 134.51, 131.74, 128.09, 128.06, 128.02, 120.55, 119.50, 117.77, 110.65. MS *m/z* 615.3 [*M*+H]^+^. Anal. calcd for C_26_H_18_Br_2_N_2_O_2_S_2_: C, 50.83; H, 2.95; N, 4.56; S, 10.44; found: C, 50.70; H, 3.01; N, 4.63; S, 10.51.

#### 2.2.8. 2-((Z)-((2-((2-(((Z)-2-hydroxybenzylidene)amino)phenyl)disulfaneyl)phenyl) imino)methyl)-6-methoxyphenol) (6)

Bright orange; yield 89%, mp: 154 °C. FTIR *ν*_max_ 2841, 1611, 1456, 1249, 749, 555 cm^−1^. ^1^H NMR (400 MHz,CDCl_3_): *δ* 3.90 (s, 3H, OCH_3_), 7.04–6.83 (m, 6H, Ar-H), 7.19–7.10 (m, 6H, Ar-H), 7.36 (t, *J* = 7.3 Hz, 2H, Ar-H), 7.66–7.59 (m, 2H, Ar-H), 8.56 (d, *J* = 2.6 Hz, 2H, H-C=N), 12.87 (d, *J* = 1.9 Hz, 1H, O-H), 13.24 (s, 1H, O-H). ^13^C NMR (101 MHz, CDCl_3_): *δ* 162.77,161.16, 151.24, 148.52, 146.21, 133.69, 132.71, 131.84, 131.58, 127.87, 127.78, 127.74, 127.64, 127.59, 127.13, 127.00, 124.13, 119.28, 119.19, 119.15, 118.86, 117.66, 117.63, 117.53, 117.41, 115.20, 56.19. MS *m/z* 487.6 [*M*+H]^+^. Anal. calcd for C_27_H_22_N_2_S_2_O_3_: C, 66.64; H, 4.56; N, 5.76; S, 13.18; found: C, 66.75; H, 4.52; N, 5.69; S, 13.24.

### 2.3. Pharmacology

#### 2.3.1. Test microorganisms

All test microorganisms are clinical isolates, obtained from the Düzce University Faculty of Medicine. The antimicrobial activities were evaluated against Gram (–) bacteria (*Acinetobacter baumannii, Escherichia coli* and *Klebsiella pneumoniae*), Gram (+) bacterium (*Staphylococcus aureus*) and yeast cultures (*Candida tropicalis*, *Candida guilliermondii*, *Candida albicans* and *Candida glabrata*) using both the disc diffusion and dilution methods [24].

#### 2.3.2. Disc diffusion method

Screening for antibacterial and antifungal activities was carried out using sterilized antibiotic discs (6 mm in diameter) (Bioanalyse) and the procedural performance standards for antimicrobial disc susceptibility tests were followed, in accordance with the Clinical and Laboratory Standards Institute (CLSI) guidelines (CLSI 2020) [25]. Fresh stock solutions (30 μg/mL^−1^) of the compounds were prepared in freshly deionized water according to the concentrations required for the experiments. Sterilized antibiotic discs (6 mm in diameter) (Bioanalyse) were impregnated with 50 mL of these solutions. All the bacteria were seeded into Difco Nutrient Broth and incubated at 30 °C for 24 h. The yeasts were incubated in Sabouraud Dextrose Agar (Oxoid) for 48 h. Inoculums containing 10^6^ bacterial cells or 10^8^ yeast cells per ml were spread on Mueller Hinton Agar (Oxoid) plates (1 mL inoculum for each plate). The solution-injected discs were lightly pressed onto the inoculated agar and incubated at 35 °C for 24 h for the bacteria and at 25 °C for 72 h for the yeast. Depending on the test microorganism, an appropriate reference antibiotic disc was applied onto each plate. Triplicate tests were performed for each case.

#### 2.3.3. Microdilution assay

All test microorganisms were obtained from various clinical samples sent to the Medical Microbiology Laboratory of the Düzce University Faculty of Medicine. The antimicrobial activities of all the compounds were determined via the broth microdilution method (CLSI 2011) [26]. The isolates were adjusted to McFarland’s 0.5 standard and 100 μL of Mueller-Hinton Broth was placed in each well of a 96-well plate. Afterwards, serial dilutions of the compounds were carried out. The concentrations of the compounds, ranging from 128 to 2 μg/mL^−1^, were calculated in order to determine the minimum inhibitory concentration (MIC). The lowest concentrations of antimicrobial agents resulting in complete inhibition of the microorganisms (mg/mL^−1^) represented the MIC. Finally, the MIC values of all compounds (2–6) against all test microorganisms were compared with standard antibiotic MIC values. For each case, triplicate tests were performed and the average was taken as the final reading.

#### 2.3.4. Cell culture

The B16F10 cell line used for the study is a malignant melanoma type with adhesive properties in the morphological epithelial cell structure. These cells were obtained from the İstanbul University Aziz Sancar Institute of Experimental Medicine, where they were cultured in accordance with ATCC protocol and stored at –80 °C. Dulbecco’s Modified Eagle’s Medium (DMEM) supplemented with 10% fetal bovine serum (FBS), 1% penicillin-streptomycin and 0.22 NaHCO_3_ was used for the subculture. Using T75 cell culture flasks, the cells were incubated in the growth medium at 37 °C in 5% CO_2_ for 60–72 h. After reaching 80% confluency in the culture flask, the cells were ready for analysis.

#### 2.3.5. Antiproliferative activity

Cell antiproliferative activity is closely related to plasma membrane damage and can be detected by several different methods. The MTT test is a colorimetric method for measuring antiproliferative activity and involves dissolving the fixated dye in appropriate solutions and then measuring them photometrically at OD 490 nm. The obtained OD value is directly proportional to the total cell content and the number of viable cells. The half maximal inhibitory concentration is a measure of the effectiveness of a compound in inhibiting biochemical processes and biological functions. According to the in vitro MTT assay, the IC50 represents the concentration of the tested agent that is required for 50% inhibition of the cell viability. IC50 is commonly used as a measure of antagonist drug potency in pharmacological research. The inhibitor concentration against the percent activity is plotted ([I]-Activity % MTT graph). Using the linear (y = mx + n) or parabolic (y = ax^2^ + bx + c) equation for y = 50 value x point becomes IC50 value.

The cultured cells were pipetted into a 96-well plate, with each well containing 3 × 10^4^ cells in 0.1 mL of medium. Eight different test molecules previously dissolved in DMEM were added to the cells at log concentrations of 0–250 μg/mL and incubated for 24 h. During the incubation period, cell proliferation and morphological changes were monitored using an inverted microscope. Next, 50 μL of each buffer were added and the cells were incubated at +37 °C for 4 h. The contents of the wells were flushed and the cells were aspirated and washed four times with 100 μL PBS, after which 200 μL of 100 mM SDS were added to each well and incubated for 30 min in the dark at room temperature. Measurements were then taken with a spectrophotometer at 490 nm.

#### 2.3.6. Statistical analysis

Cell viability was analyzed by performing one-way analysis of variance and multiple comparison analyses were performed using SPSS software (version 21.0; IBM Corporation, Armonk, NY, USA). Each data point was repeated in three independent experiments. p < 0.05 was considered to indicate a statistically significant difference.

## 3. Results and discussion

### 3.1. Chemistry

**Figure 1:**
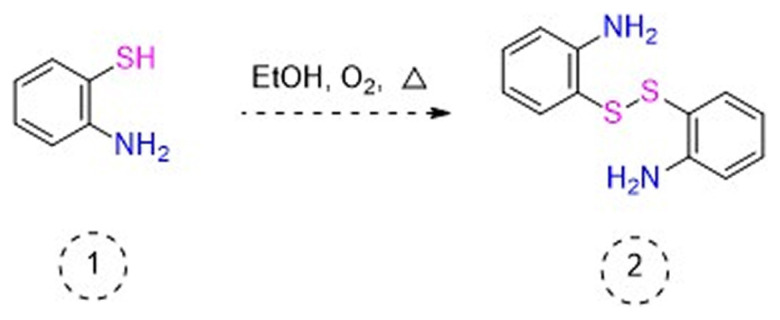
Synthesis of 2,2′-disulfanediyldianiline **(2)**.

Higher yields of the synthesized compounds were obtained using the CeO_2_ nanocatalyst. The 2,2′-disulfanediyldianiline 2 was synthesized as a starting material for dimeric disulfide-Schiff bases. The synthesis of the dimeric disulfide-Schiff bases 3a-6 was carried out by reacting compound 2 with several different aldehydes, including 2-hydroxy-3-methyl benzaldehyde, 2-hydroxy-5-methyl benzaldehyde, 2-hydroxy-3-nitro benzaldehyde, 2-hydroxy-5-nitro benzaldehyde, 4-hydroxy-3-nitro benzaldehyde, 2-hydroxy-5-bromo benzaldehyde and 2-hydroxy-3-methoxy benzaldehyde, respectively. Furthermore, the synthesis of these dimeric disulfide-Schiff bases was carried out in two different ways: one using the CeO_2_ nanocatalyst and one without the catalyst (Figure 1 and Table 1). As a result of the comparison, the results which supported the spectral data of the compounds in our study positively were obtained [27]. Functional groups such as −NH_2_, −OH, −NO_2_, CH_3_ and −OCH_3_ were found in the structures of these dimeric disulfide-Schiff base compounds. In compounds 3a and 3b, characteristic peaks belonging to the *υ*(C-CH_3_) group were observed at 2906 cm^−1^ and 2911 cm^−1^, respectively. In compound 6, the characteristic peak of the methoxy group *υ*(C-OCH_3_) was observed at 2841 cm^−1^ [28,29]. In compound 4c, the characteristic peak of the hydroxy group *υ*(OH) was observed at 3196 cm^−1^. Besides, in compounds 4a, 4b and 4c, characteristic peaks belonging to the *υ*(C-NO_2_) group were observed at 1345 cm^−1^, 1337 cm^−1^ and 1533 cm^−1^, respectively. Moreover, the sharp, extended vibrations of the *υ*(C=N) (azomethine) group, which form the foundation of the Schiff bases, were observed in the range of 1620–1609 cm^−1^ [30,31]. Specific peaks of *υ*(C=C_Ar_), *υ*(C-O_Ar_), *υ*(C-S) [32] and *υ*(S-S) were observed in the ranges of 1487–1428, 1292–1249, 754–735 and 595–555 cm^−1^ [33,34], respectively. At the same time, in compound 5, the characteristic peak of the *υ*(C-Br) group was observed at 624 cm^−1^. The mass spectra of compounds 2–6 further validated the structures of the synthesized compounds. The ^1^H NMR and ^13^C NMR spectra of the synthesized compounds were recorded using DMSO-*d_6_* and CDCl_3_ solvents. When investigating the ^1^H NMR spectra of the compounds, there were some specific proton signals that needed to be addressed. The protons of the hydroxy, methoxy and methyl functional groups in the structure of the compounds were recorded as a singlet with chemical shifts of 13.85–10.90, 3.90 and 2.33 ppm, respectively. The signals of the azomethine (H-C=N) proton were recorded as a singlet proton at 8.70–8.53 ppm [35]. Proton signals belonging to the aromatic ring in the structures of the synthesized compounds were found as doublets, triplets and multiplets in the range of 6.56–8.46 ppm. With both methods, all compounds were obtained in a highly efficient manner; nevertheless, in the method using the CeO_2_ nanocatalyst, the yield was significantly greater compared with that of the method using no catalyst. At the same time, the method in which CeO_2_ nanocatalyst is used makes a positive contribution to the literature as a different and innovative synthesis method, as it significantly reduces the reaction time compared to the methods used in previous studies (Table 2) [34,36].

### 3.2. Pharmacological data

#### 3.2.1. Antimicrobial screening

The synthesized compounds were screened for antibacterial and antifungal activity against the following organisms: Gram (–) bacteria (*Acinetobacter baumannii*, *Escherichia coli* and *Klebsiella pneumoniae*), Gram (+) bacteria (*Staphylococcus aureus*) and Candida species (*C. tropicalis*, *C. guilliermondii*, *C. albicans* and *C. glabrata*). This was done using the disc diffusion and microdilution methods following standard procedures and the results are given in Tables 3 and 4. Antimicrobial effects of synthesized compounds were compared with reference drugs (Cefotaxime, Amoxicillin/Clavulanic acid Posaconazole, and Nystatine). According to disc diffusion results, all the tested compounds showed varying inhibition zones against the pathogens. The results revealed that compound **2** showed the maximum activity against *K. pneumoniae* (21 mm), with significant activity against other bacteria. In addition, compound **2** showed significant activity against *Candida guilliermondii* (25 mm). Among all the compounds, compound **3a** showed the least activity for bacterial and fungal isolates (6–12 mm) when compared to standard antibiotics. Abou-Hussein and Wolfgang were evaluated the Schiff base, HLa-Maf and H2Lb-Daf and their metal complexes for antimicrobial activity against Gram-positive bacteria (*Staphylococcus aureus* and *Pseudomonas fluorescens*), Gram-negative bacteria and pathogenic fungus. Researchers showed that these compounds have strong antibacterial activity (16–40 mm) and antifungal activity (16–36 mm) [37]. According to Sabry Hamed and Abdel Aziz, the antimicrobial activity of the Schiff base ligand and its metal complexes were tested against bacteria (*Staphylococcus aureus*, *Pseudomonas aeruginosa*, *Escherichia coli*, *Staphylococcus epidermidis*) and fungi (*Aspergillus niger*, *Aspergillus flavus*, *Curvularia lunata* and *Candida albicans*). They have revealed that Schiff base complexes have the same effect with commercial antibiotic. Our findings are consistent with these results [38].

According to the MIC results, apart from a few exceptions, all the compounds were more active against all the tested microorganisms compared to antibacterial and antifungal antibiotics. In general, all compounds were similarly active against the test microorganisms, except for compound **2**. However, compound **2** exhibited good activity against bacteria and fungi when compared to standard antibiotics. The compound **3a** activity toward *A. baumannii* and *K. pneumoniae* was equipotent to that of gentamycin, with doses of over 64 μg/mL of the compound needed for the inhibition of these microorganisms. The results of this study indicated that these compounds have the potential to generate novel metabolites for developing new antibiotics. Their strong effect on bacteria and fungi and particularly their lethal antibacterial activity could result in the formulation of novel drugs. These compounds should be singled out for further pharmacological studies to evaluate their potential use against various infectious diseases [39].

#### 3.2.2. Anticarcinogenic effect of Schiff bases on melanoma cells

To determine the antiproliferative effect of synthesized compounds containing Schiff bases, all of these eight compounds were applied separately on B16F10 cells at doses of 0–250 μM for 24 h. Our results revealed that all of these compounds exhibit variable levels of antiproliferative activity. According to our results, the first 4 compounds; compound **2, 3a**, **3b** and **4a** showed a similar antiproliferation profile. At 12.5 μM, the lowest dose application of these first four compounds, the viability of the cells showed a drastic reduction of about 60% compared to control cells which are not treated with any compound. At subsequent doses, there was no significant change observed except for somewhat proliferative effect of compound **3a** at the dose value of 250 μM. Our most interesting finding, among the compounds we examined, was the effects after compound **4c** administration. This compound showed the lowest antiproliferative response to the first dose of 12.5 μM. Because the viability of cells still seemed just over 80%. However, compared to all compounds, we observed the lowest cell viability only in compound **4c** at 37.5 μM dose application. Viability of cells at this dose was approximately 48%. At the higher concentration applications of compound **4c**, there was no significant change in the viability of the cells. According to our results, the least toxic compound was compound **4b**. In compound **5** application, cells showed a linear antiproliferation profile. When we look at the results of all the compounds we apply to the cells, it was seen that the decrease in the viability of the cells were significantly up to 37.25 μM dose. We did not observe a significant change in cell viability at doses above this value. It was also seen that the cells were separated from each other and lose their integrity, probably due to the breaking of the adhesive bonds. Thus, in general, after administration of the compounds, the morphological phenotypes of cells, including shape, size and density were observed to change in response. All compounds showed a statistically significant decrease in proliferation level compared to control group cells at all application doses (p < 0.05). Quantitative values of mean cell viability (%) with standard deviations after administration of compounds are shown in Table 5.

**Figure 2:**
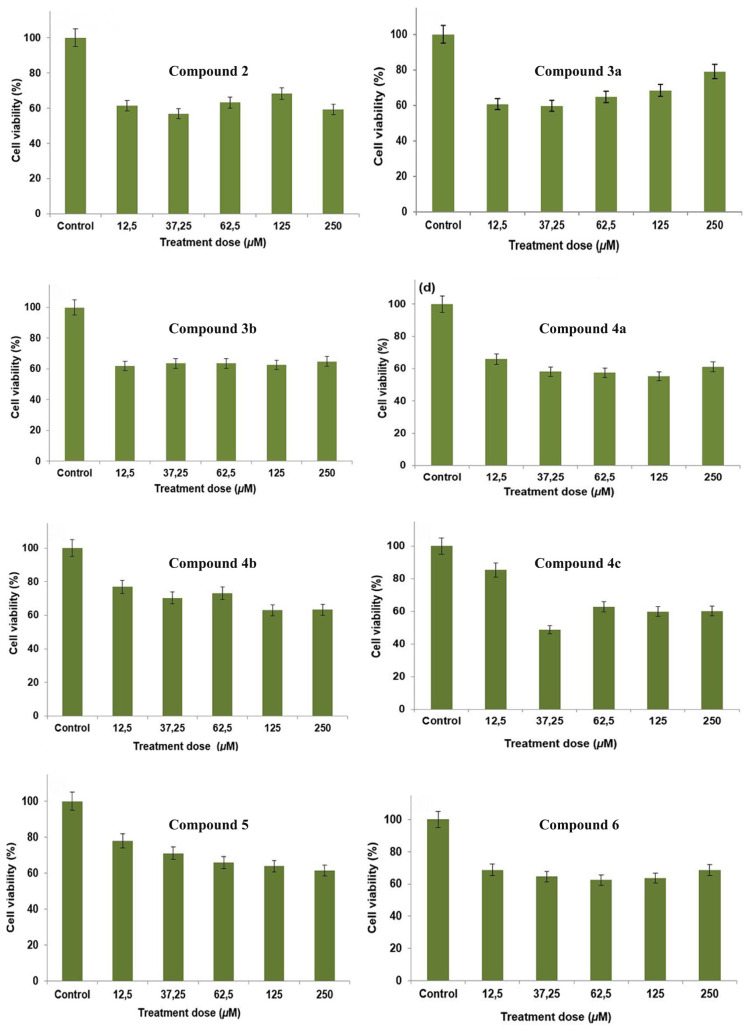
Microscopic view of morphological changes that occurred in B16F10 cells as a result of applying all synthesized compounds separately for 24 h and at a concentration of 250 μM.

**Figure 3:**
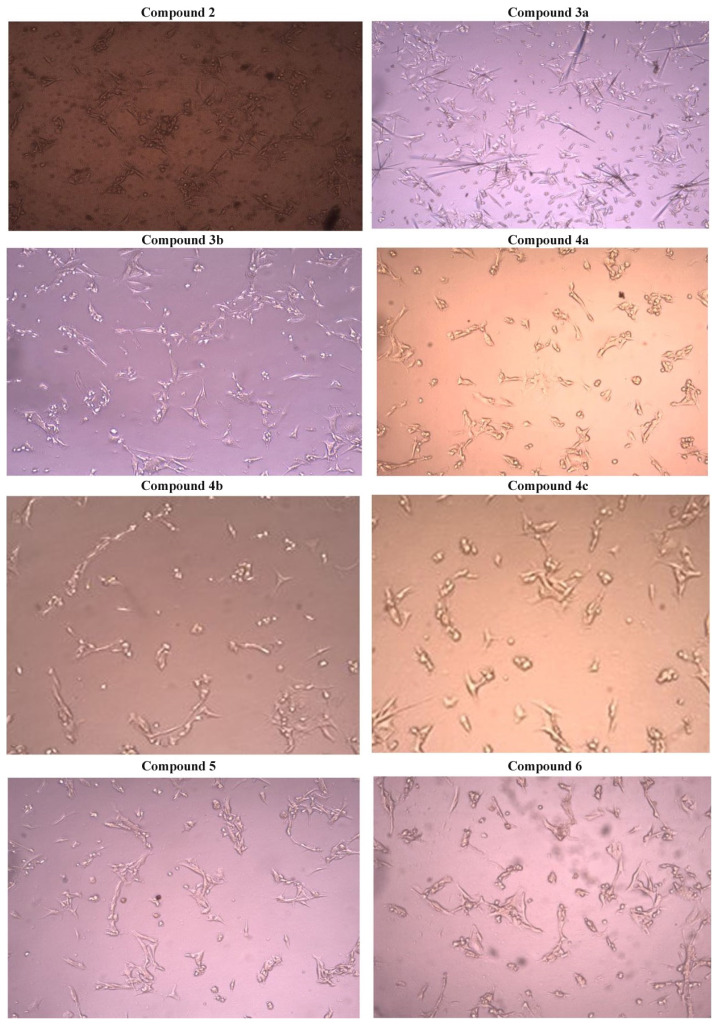
The effect of all synthesized compounds on cell viability in B16F10 melanoma cells. Cells were treated separately with these compounds at 0–250 μM concentration levels for 24 h.

After the incubation period, the effect of these compounds on cell proliferation and morphological changes was observed with inverted microscope (Figure 2). It also was an interesting finding to see that changes in the chemical structure of these Schiff bases-compounds could significantly affect antitumor activities [40,41]. The complexes had specific effects; for instance, they could induce apoptosis in cancer cells or they were antiproliferative agents with high selectivity (Figure 3). Morphological changes revealed that, compounds **3a** and **4a** in the ortho-meta position to each other had the greatest effect on cancer cells compared to other compounds in the ortho-para position. Moreover, compound **2** showed anticancer activity at a similar level with compounds **3a** and **4a** due to the functional groups it contained. Asadizadeh et al. showed that Schiff base ligands have potent antiproliferative effect against two human tumor cell lines (HeLa and MCF-7). They showed that some compounds are highly potent against the MCF-7 cell line than clinically used cisplatin, so that they may have the potential to act as effective metal-based anticancer drugs [42]. John et al. obtained promising findings with highly toxic effects as a result of the application of metal Schiff base complexes to PA1 ovarian cancer cells. In our study, compounds applied to B16F10 melanoma cell lines showed a proliferation inhibition rate varying between 20% and 40% at the lowest application dose of 12.5 μM, which was further increased at higher doses [43].

## 4. Conclusion

The compounds were obtained in a much shorter time via a CeO_2_ nanocatalyst. This method is practical, inexpensive, easy and environmentally friendly, and the reaction takes minutes instead of hours. The 2,2′-disulfanediyldianiline was derivatized with various aldehydes to obtain seven dimeric disulfide-Schiff base compounds **(3a-6)**. Looking at all these results, different compounds can be studied so that the skeletal structure remains the same. The strong antimicrobial results of the synthesized compounds, especially compound 2 (17–25 mm inhibition zones), on test microorganisms showed that these compounds have potential for the development of formulations of new antibiotics. In addition, the high antiproliferative activity of the synthesized compounds at lowest dose (12.5 μM) on the B16F10 melanoma cell line, may lead to the use of these compounds as a potential group of anticancer agents, after further confirmation by detailed in vivo experiments. However, further pharmacological studies are required to evaluate the potential uses of these compounds.

## Supplementary information

Figure S1 Compound 2 FTIR spectrum.

Figure S2 Compound 3a FTIR spectrum.

Figure S3 Compound 3b FTIR spectrum.

Figure S4 Compound 4a FTIR spectrum.

Figure S5 Compound 4b FTIR spectrum.

Figure S6 Compound 4c FTIR spectrum.

Figure S7 Compound 5 FTIR spectrum.

Figure S8 Compound 6 FTIR spectrum.

Figure S9 Compound 2 ^1^H NMR spectrum.

Figure S10 Compound 3a ^1^H NMR spectrum.

Figure S11 Compound 3b ^1^H NMR spectrum.

Figure S12 Compound 4c ^1^H NMR spectrum.

Figure S13 Compound 5 ^1^H NMR spectrum.

Figure S14 Compound 6 ^1^H NMR spectrum.

Figure S15 Compound 2 ^13^C NMR spectrum.

Figure S16 Compound 3a ^13^C NMR spectrum.

Figure S17 Compound 5 ^13^C NMR spectrum.

Figure S18 Compound 6 ^13^C NMR spectrum.

Figure S19 Compound 2 MS spectrum.

Figure S20 Compound 3b MS ^s^pectrum.

Figure S21 Compound 4a MS spectrum.

Figure S22 Compound 4b MS spectrum.

Figure S23 Compound 4c MS spectrum.

Figure S24 Compound 6 MS spectrum.

## Figures and Tables

**Table 1:** Dimeric disulfide-Schiff base compounds derived from 2,2′-disulfanediyldianiline.

Entry	Amines	Aldehydes	Product
3a	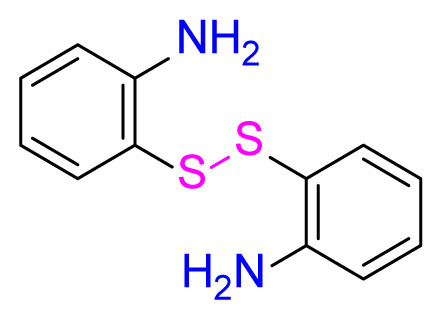	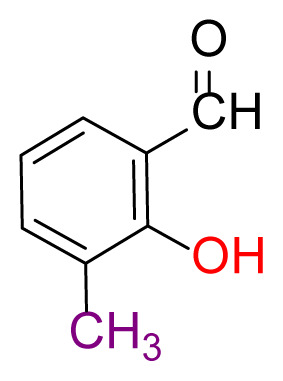	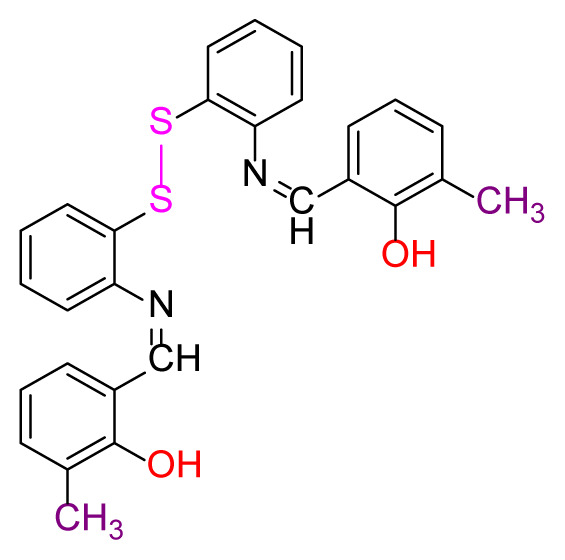
3b	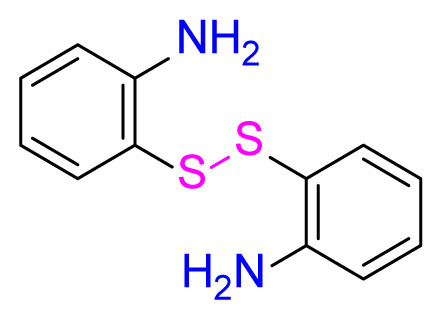	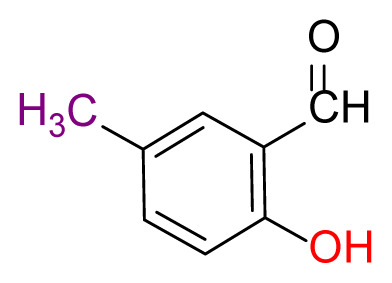	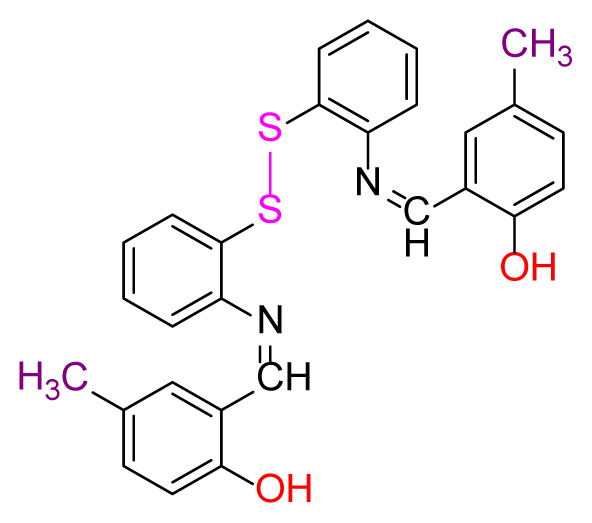
4a	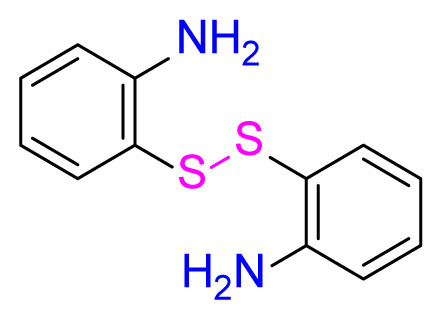	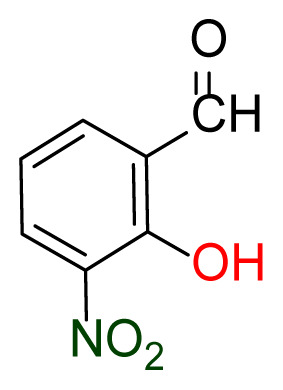	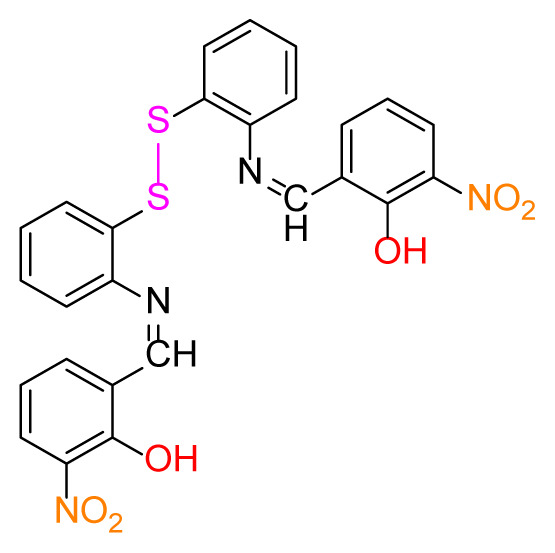
4b	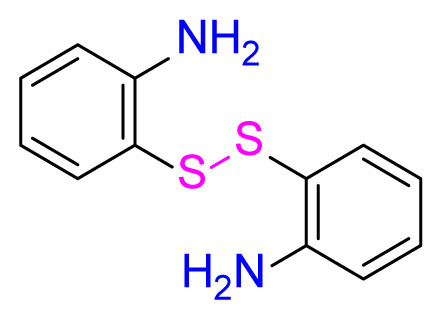	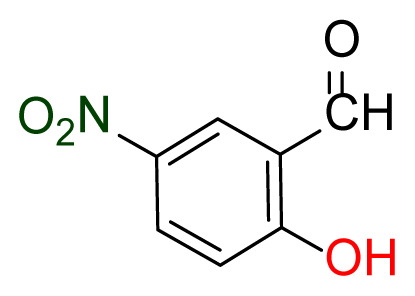	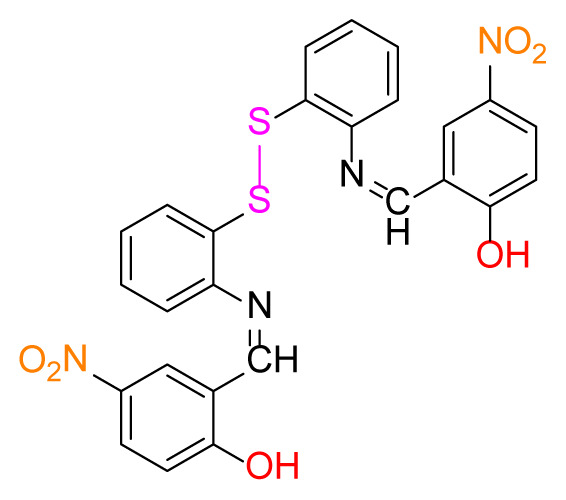
4c	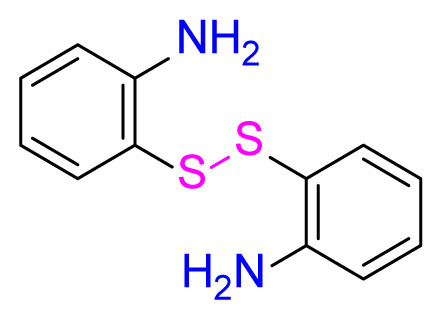	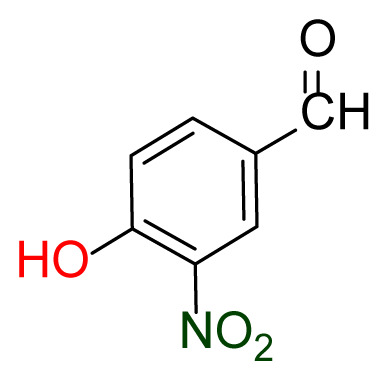	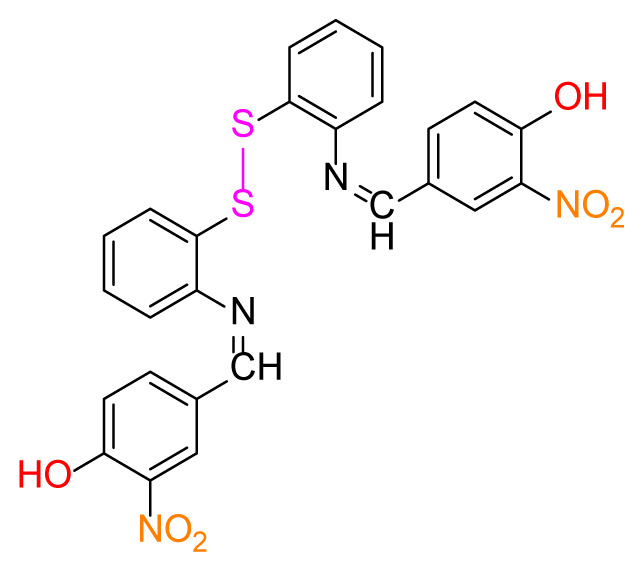
5	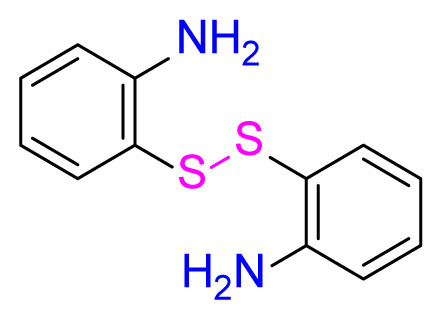	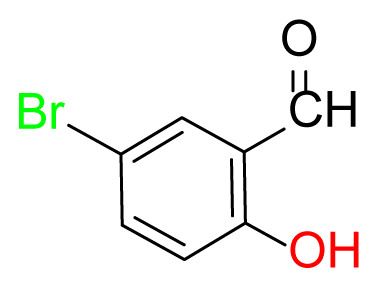	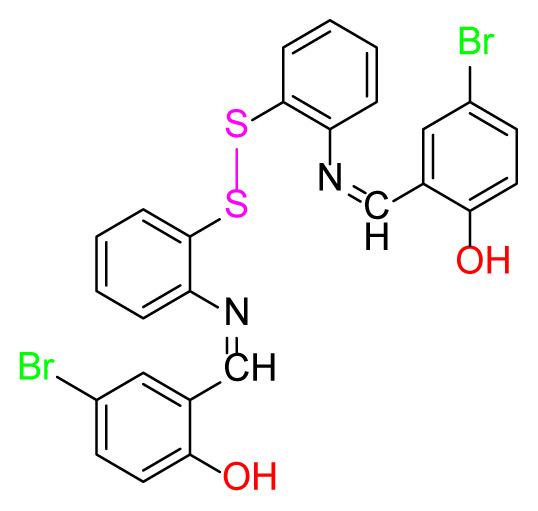
6	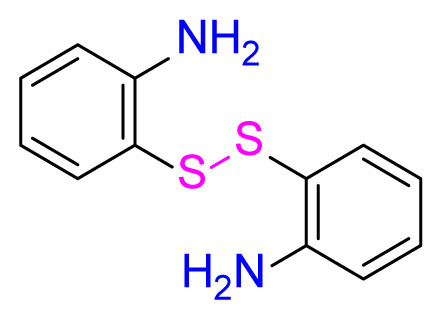	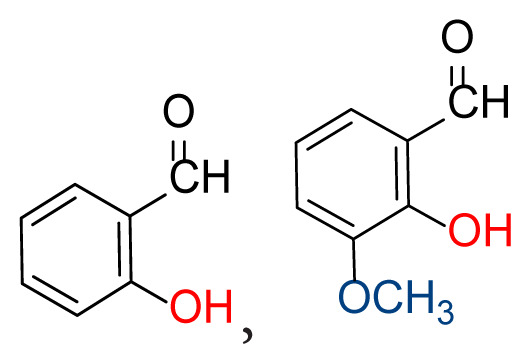	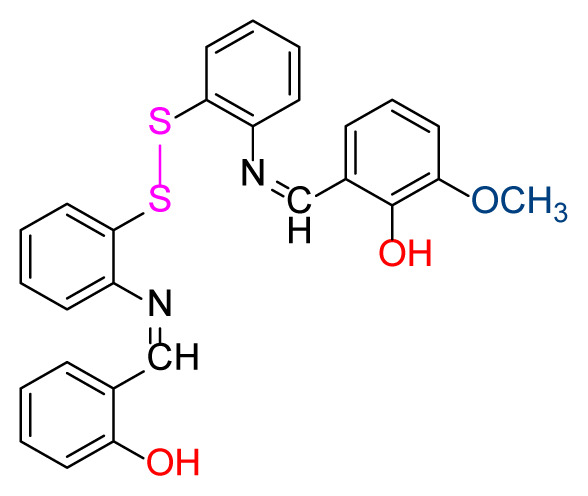

**Table 2:** Reaction parameters (reaction time, yield) of synthesized dimeric disulfide-Schiff base compounds.

Entry	Reaction time		Yield (%)	
	Without CeO_2_ nanocatalyst	CeO_2_ nanocatalyst	Without CeO_2_ nanocatalyst	CeO_2_ nanocatalyst
	(h)	(min)		
2	9	-	75	-
3a	4	10	82	90
3b	5	15	84	93
4a	6	15	87	100
4b	6	15	89	97
4c	6	15	83	95
5	6	15	75	95
6	7	15	73	89

**Table 3:** The in vitro antimicrobial activity of compounds **2–6**.

Inhibition zones (mm)*												
CompoundsAntibiotics												
Gram-negative bacteria	2	3a	3b	4a	4b	4c	5	6	CTX30	AMC30	PCZ5	NY100
*Acinetobacter baumanni*i	20.0 ± 1.8	7.0 ± 0.0	10.0 ± 0.5	11.0 ± 1.0	9.0 ± 0.5	11.0 ± 0.3	8.0 ± 0.4	11.0 ± 0.1	7.0 ± 0.0	11.0 ± 0.4	-	-
*Escherichia col*i	20.0 ± 1.0	12.0 ± 09	11.0 ± 0.2	10.0 ± 0.6	10.0 ± 0.2	12.0 ± 0.4	7.0 ± 0.0	10.0 ± 0.0	6.0 ± 0.0	13.0 ± 0.1	-	-
*Klebsiella pneumonia*e	21.0 ± 1.1	8.0 ± 0.5	9.0 ± 0.0	10.0 ± 0.5	11.0 ± 0.5	12.0 ± 0.3	7.0 ± 0.0	11.0 ± 0.0	6.0 ± 0.0	13.0 ± 0.0	-	-
**Gram-positive bacterium**												
*Staphylococcus aureu*s	17.0 ± 1	15.0 ± 1	10.0 ± 0.9	8.0 ± 0	14.0 ± 0	9.0 ± 0.5	10.0 ± 0.9	11.0 ± 0.3	8.0 ± 0.5	10.0 ± 0.3	-	-
***Candida*** ** species**												
*Candida tropicali*s	20.0 ± 0.2	6.0 ± 0.0	8.0 ± 1.0	11.0 ± 0.4	12.0 ± 0.0	12.0 ± 0.3	7.0 ± 0.0	6.0 ± 0.0	-	-	12.0 ± 0.0	7.0 ± 0.0
*Candida guilliermondii*	25.0 ± 1.0	9.0 ± 1.0	9.0 ± 0.2	10.0 ± 0.0	11.0 ± 0.2	16.0 ± 0.1	11.0 ± 0.3	11.0 ± 0.5	-	-	13.0 ± 0.1	7.0 ± 0.2
*Candida albicans*	20.0 ± 0.3	8.0 ± 0.5	10.0 ± 0.9	13.0 ± 0.0	10.0 ± 0.7	12.0 ± 0.8	8.0 ± 1.0	12.0 ± 0.8	-	-	20.0 ±0.4	8.0 ± 0.0
*Candida glabrata*	17.0 ± 0.2	7.0 ± 0.0	8.0 ± 1.0	12.0 ± 0.0	10.0 ± 0.6	11.0 ± 0.5	7.0 ± 0.0	9.0 ± 0.0	-	-	19.0 ± 0.1	10.0 ± 0.5

CTX30: Cefotaxime 30 μg; AMC30: Amoxicillin/clavulanic acid 30 μg; PCZ5: Posaconazole 5 μg; NY100: Nystatine 100 μg. All compound doses: 30 μg. (*). The figures on the scale show the inhibition diameters.

**Table 4:** The in vitro antimicrobial activity (MIC, μg/mL^−1^) of compounds **2–6**.

Ligands									Antibiotics	
Gram-negative bacteria	2	3a	3b	4a	4b	4c	5	6	GEN30	NYS30
*Acinetobacter baumannii*	8	64	32	32	32	32	64	32	> 64	-
*Escherichia coli*	8	16	32	32	32	16	64	32	64	-
*Klebsiella pneumoniae*	8	64	32	32	32	16	64	32	32	-
**Gram-positive bacterium**										
*Staphylococcus aureus*	8	8	32	64	16	64	32	32	64	-
***Candida*** ** species**										
*Candida tropicalis*	8	64	64	32	16	16	64	64	-	16
*Candida guilliermondii*	4	32	32	32	32	8	32	32	-	32
*Candida albicans*	8	64	32	16	32	16	64	16	-	8
*Candida glabrata*	8	64	64	16	32	16	64	64	-	8

GEN30: Gentamycin 30 μg; NYS30: Nystatin 30 μg. All compound doses: 30 μg.

**Table 5:** Quantitative values of average cell viability (%) with standard deviations after administration of compounds.

Compounds								
Treatment doses (μM)	2	3a	3b	4a	4b	4c	5	6
**12.5**	59.83 ± 1.25	60.23 ± 0.56	61.46 ± 0.60	65.16 ± 0.40	76.30 ± 0.45	84.13 ± 0.45	75.56 ± 0.56	66.83 ± 0.70
**37.25**	55.06 ± 1.12	58.20 ± 1.05	63.70 ± 0.60	55.56 ± 0.98	70.76 ± 0.70	49.20 ± 0.65	70.80 ± 0.65	61.90 ± 0.20
**62.5**	63.90 ± 0.91	64.86 ± 0.86	63.80 ± 0.45	54.20 ± 0.45	73.90 ± 0.30	64.70 ± 0.75	65.23 ± 0.35	60.46 ± 0.70
**125**	63.30 ± 0.65	68.30 ± 0.80	62.43 ± 0.47	51.86 ± 0.25	64.3 ± 0.55	59.63 ± 0.75	63.13 ± 0.55	61.76 ± 0.55
**250**	60.36 ± 0.86	77.36 ± 0.96	64.33 ± 0.73	58.23 ± 0.51	65.73 ± 0.47	61.5 ± 0.60	60.50 ± 0.79	70.43 ± 0.86
**Control**	99.73 ± 1.81	101.0 ± 1.65	100.63 ± 1.10	101.00 ± 0.70	101.10 ± 0.88	101.16 ± 0.60	100.70 ± 0.50	100.83 ± 0.40
