# Supplementary figures and images for: Thio-Schiff bases derived from 2,2′-disulfanedianiline via nanocerium oxide: antimicrobial effect and antiproliferative effects in melanoma cells
Source: Turk J Chem. 2022 Mar 2;46(4):1055–68. doi: 10.55730/1300-0527.3414 (PMC10395709; doi:10.55730/1300-0527.3414)

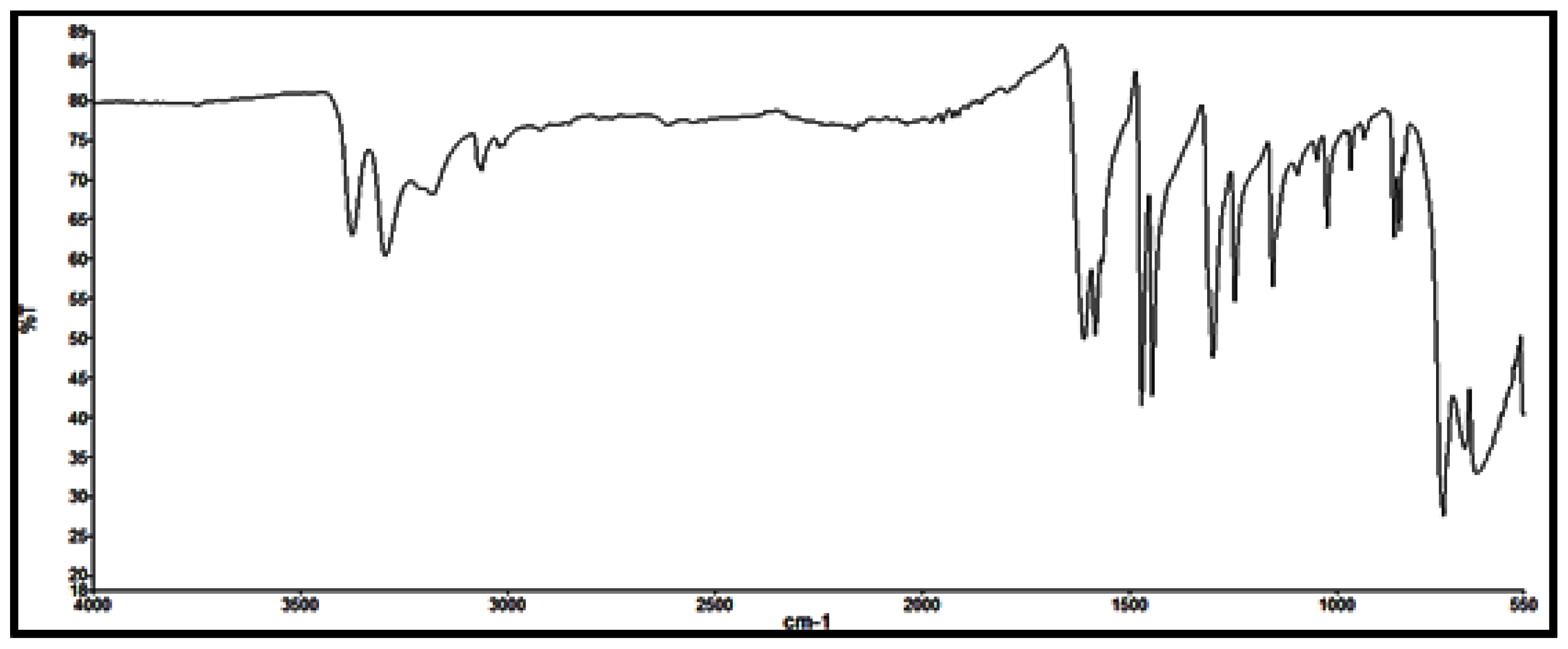

Supplement: Figure S1 — Compound 2 FTIR spectrum. [file turkjchem-46-4-1055s1.tif]

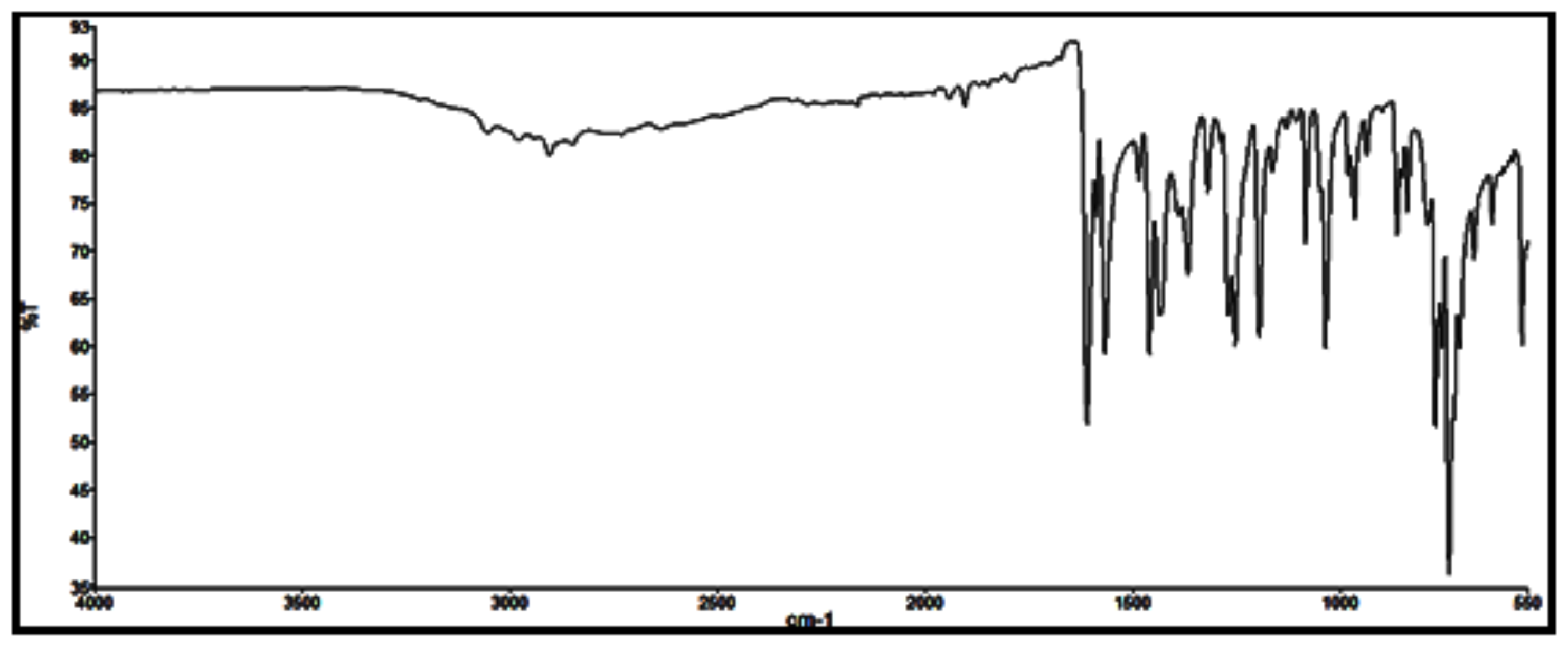

Supplement: Figure S2 — Compound 3a FTIR spectrum. [file turkjchem-46-4-1055s2.tif]

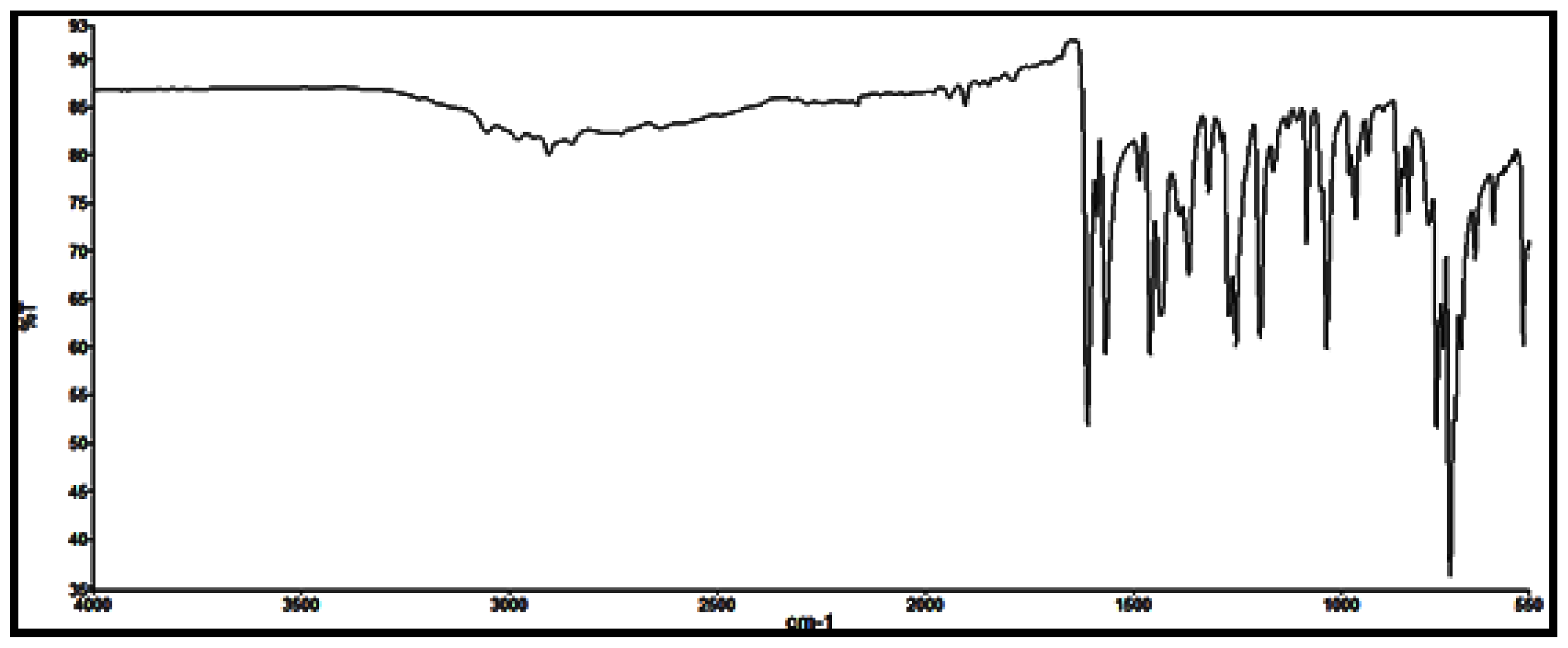

Supplement: Figure S3 — Compound 3b FTIR spectrum. [file turkjchem-46-4-1055s3.tif]

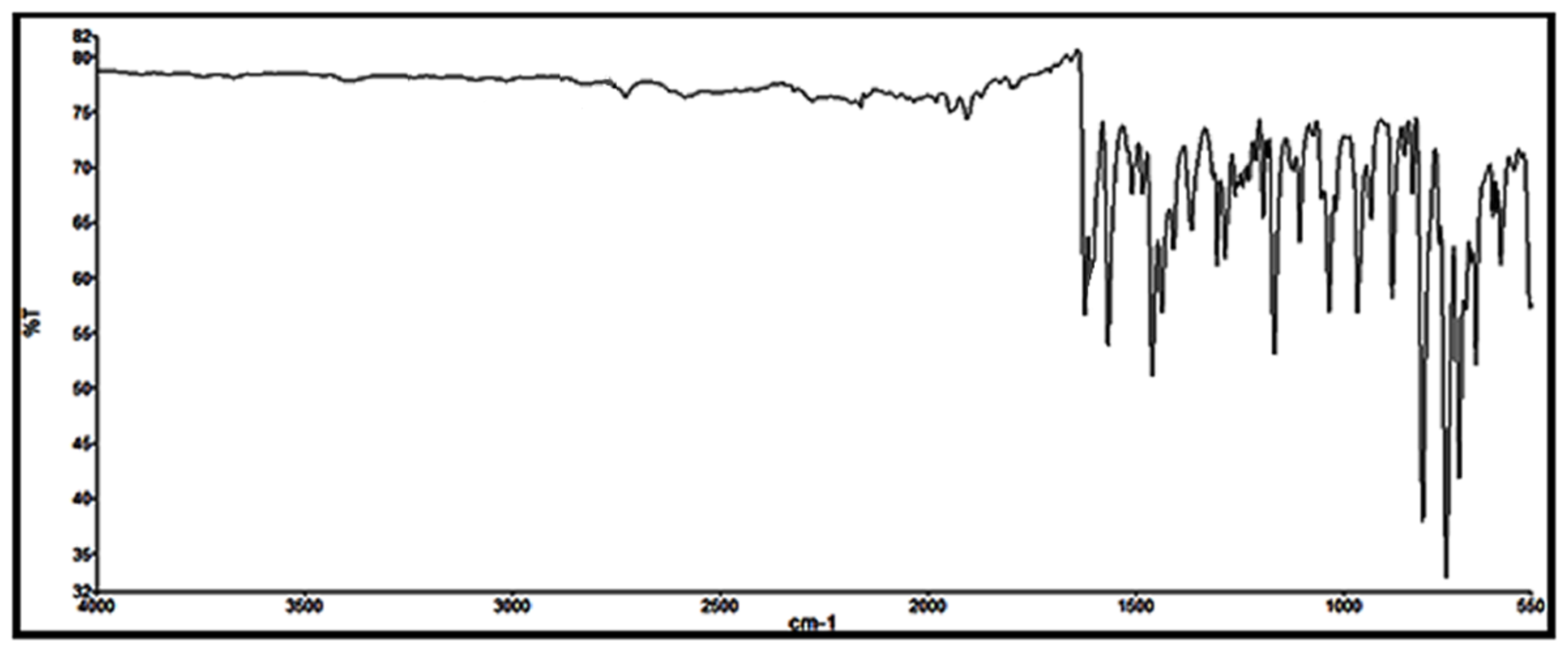

Supplement: Figure S4 — Compound 4a FTIR spectrum. [file turkjchem-46-4-1055s4.tif]

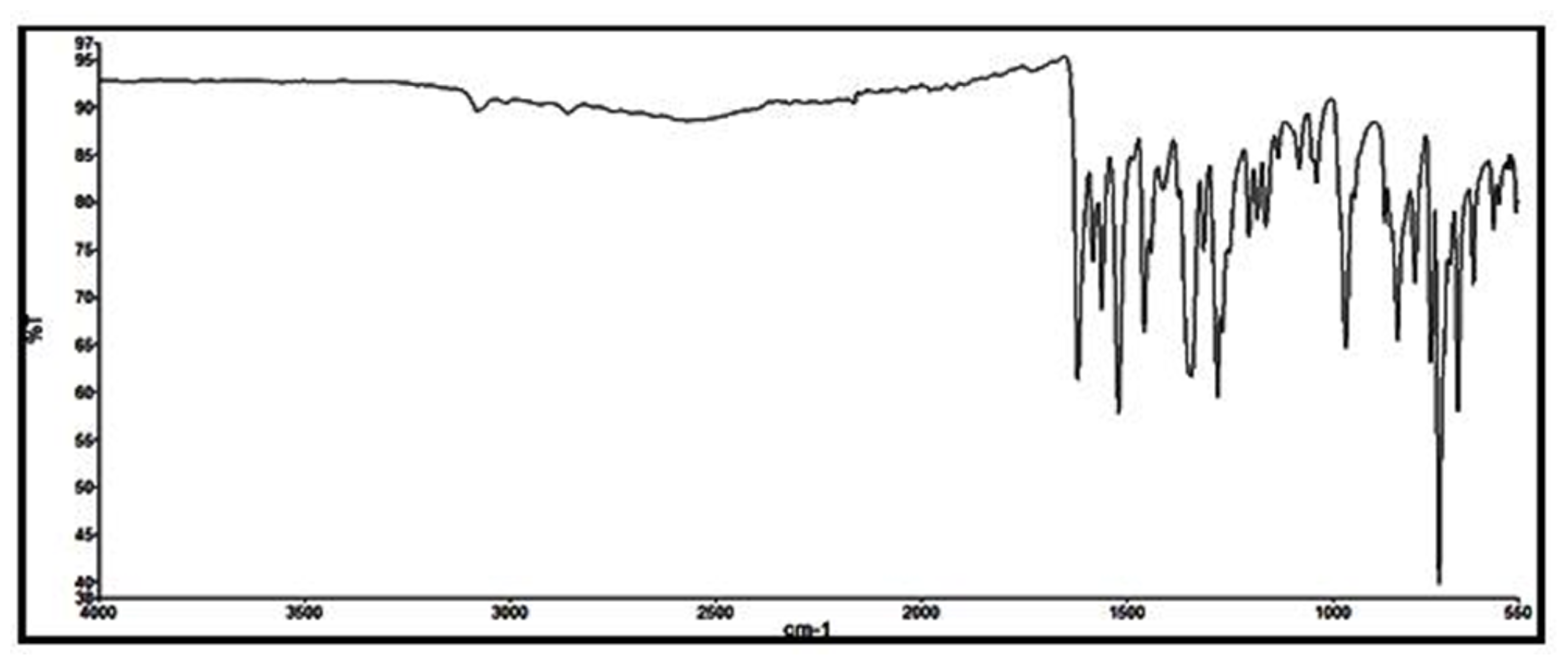

Supplement: Figure S5 — Compound 4b FTIR spectrum. [file turkjchem-46-4-1055s5.tif]

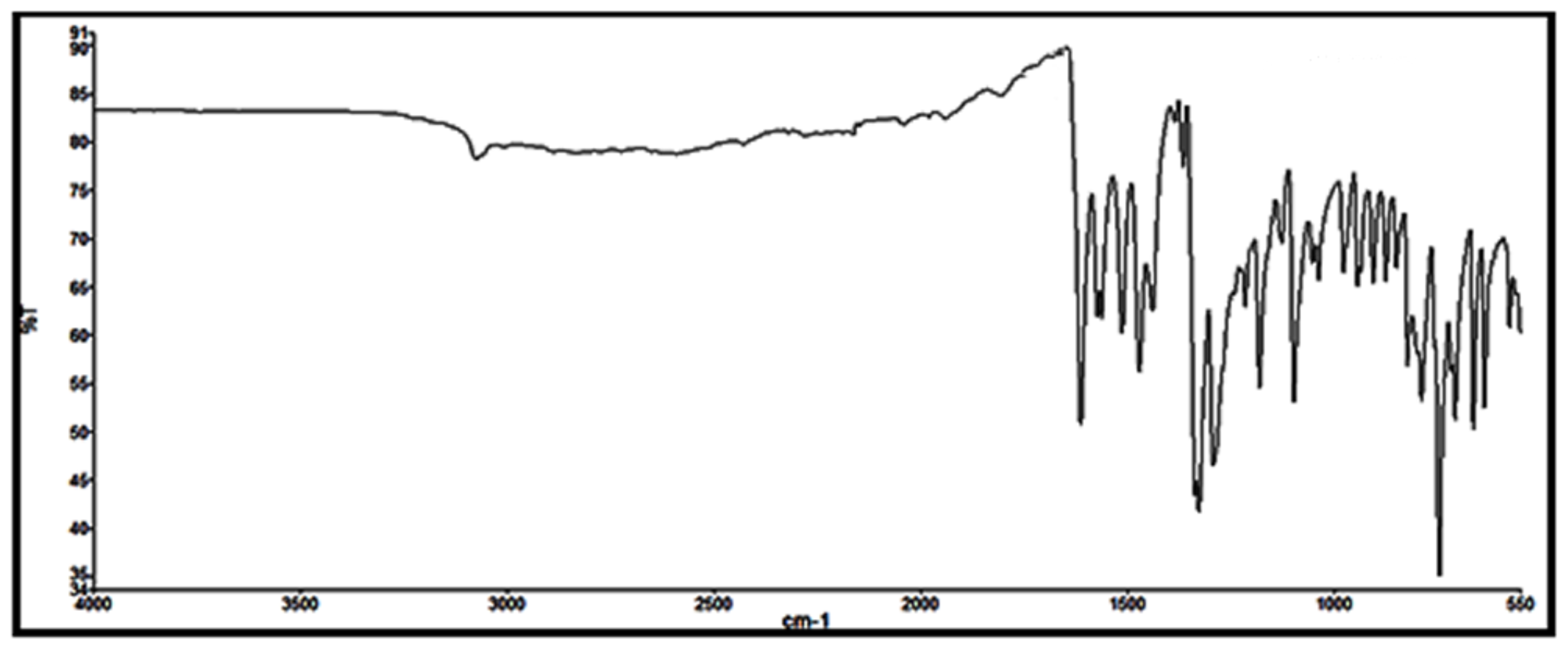

Supplement: Figure S6 — Compound 4c FTIR spectrum. [file turkjchem-46-4-1055s6.tif]

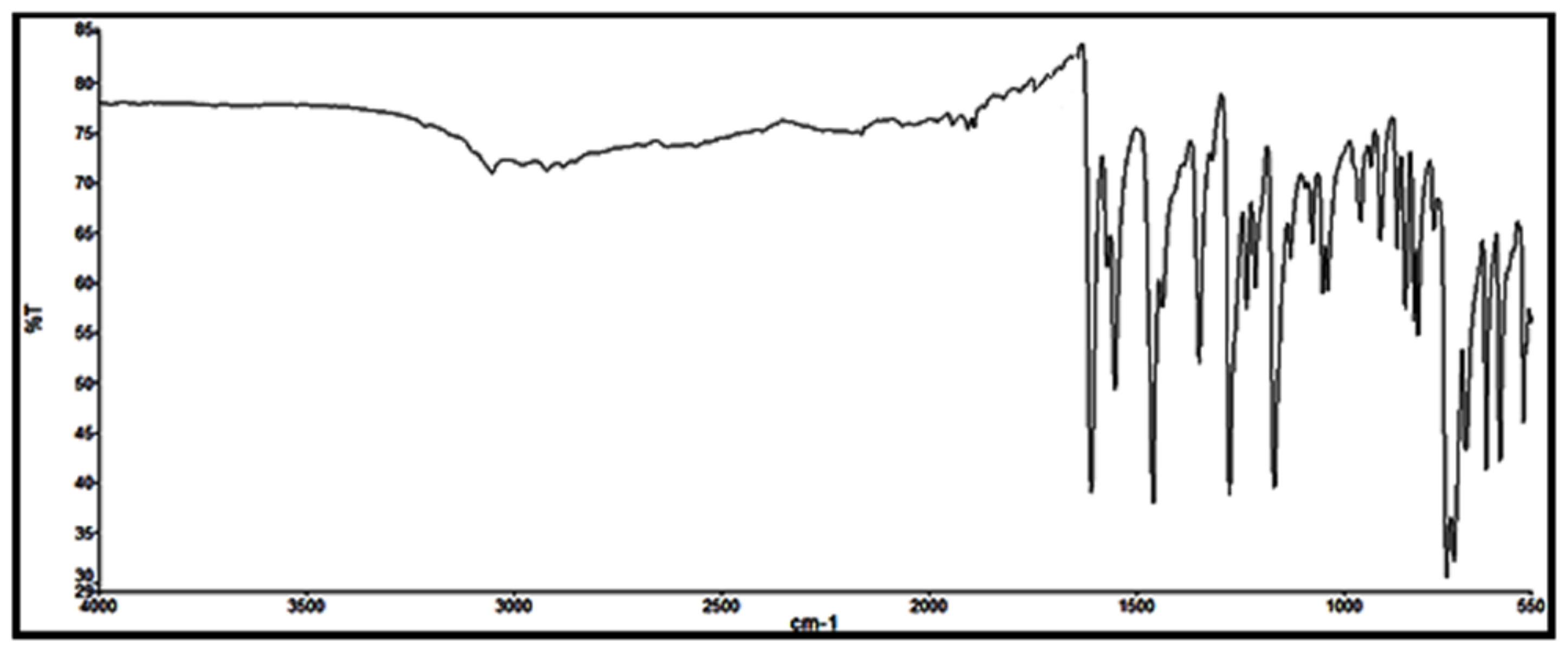

Supplement: Figure S7 — Compound 5 FTIR spectrum. [file turkjchem-46-4-1055s7.tif]

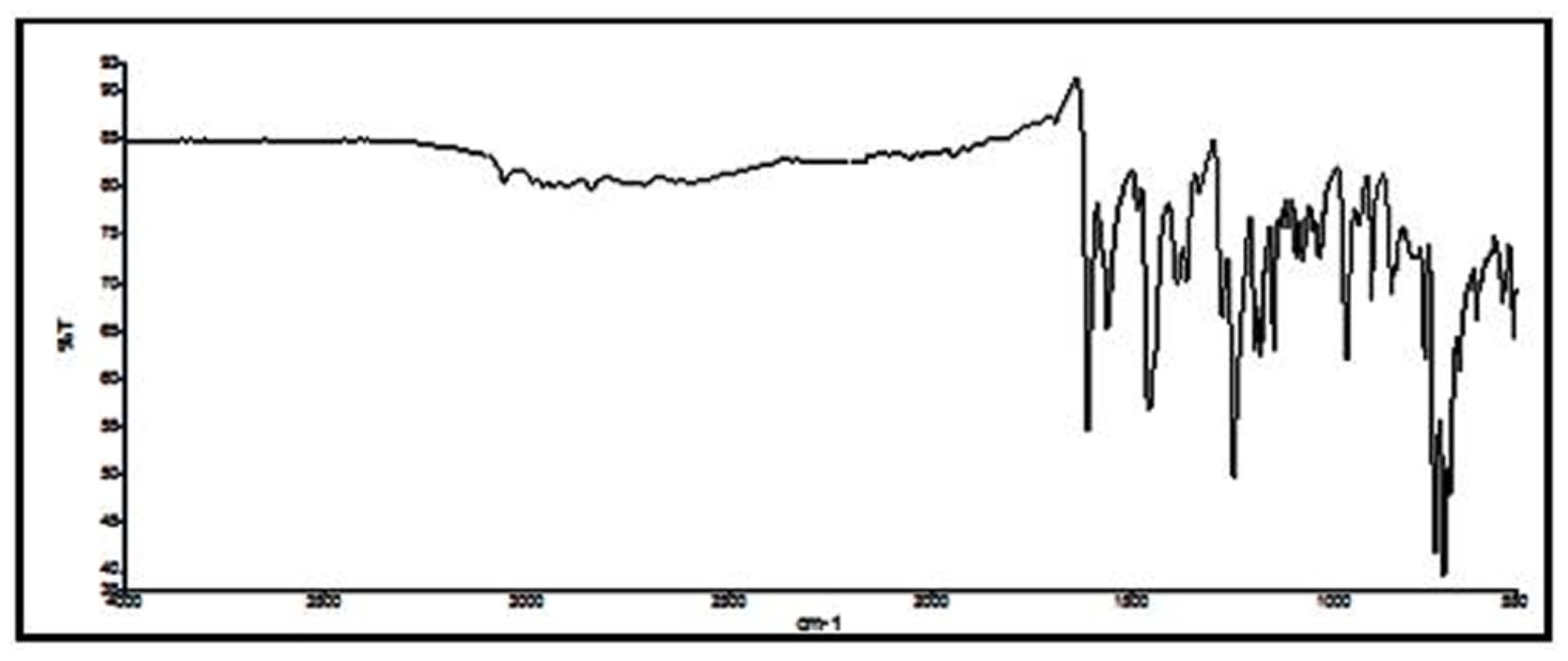

Supplement: Figure S8 — Compound 6 FTIR spectrum. [file turkjchem-46-4-1055s8.tif]

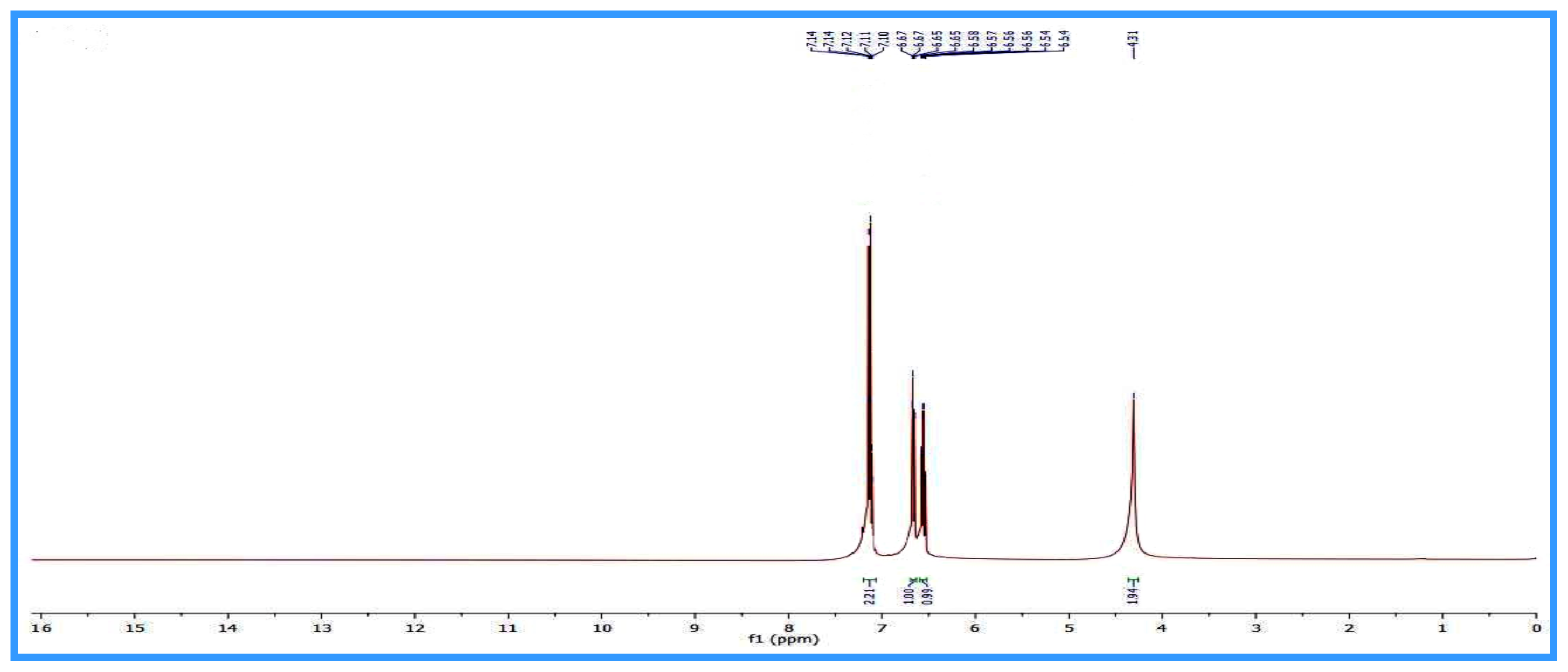

Supplement: Figure S9 — Compound 2 1H NMR spectrum. [file turkjchem-46-4-1055s9.tif]

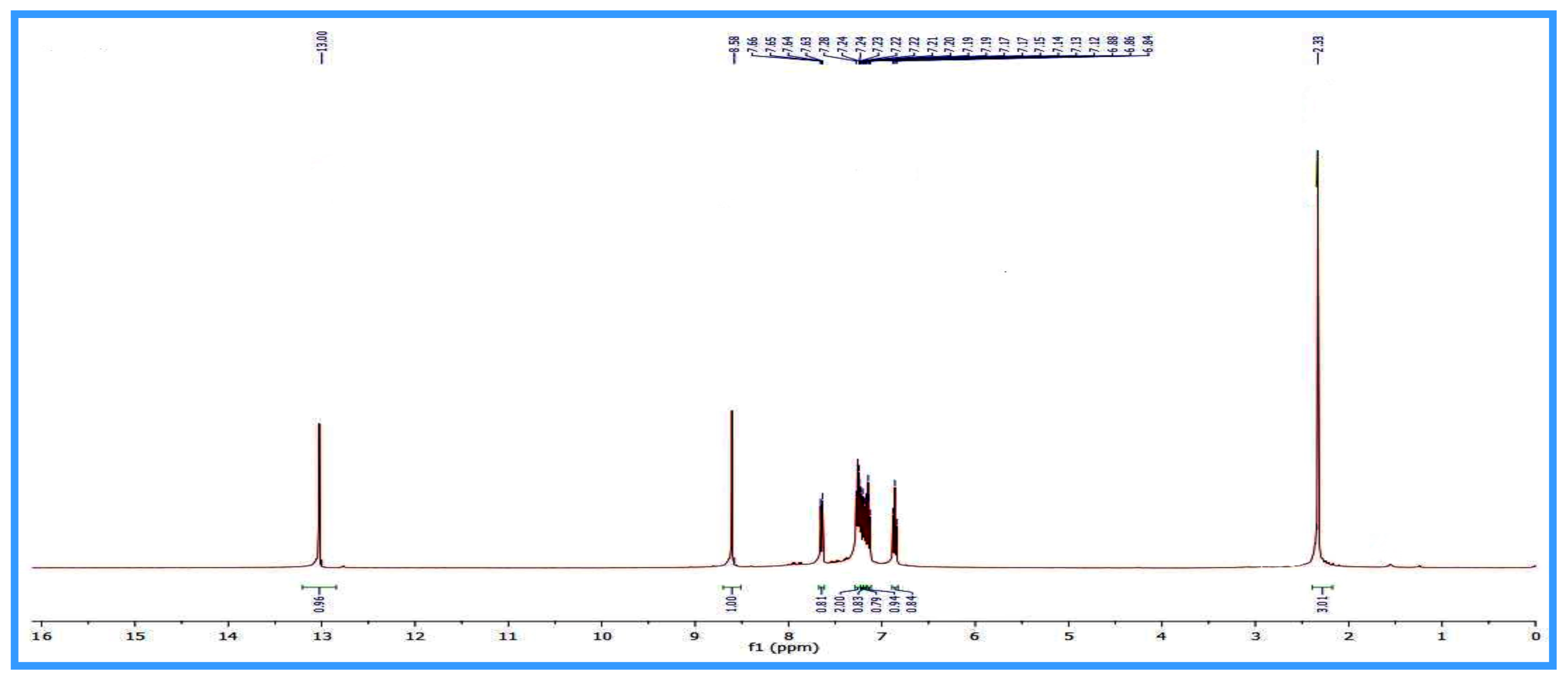

Supplement: Figure S10 — Compound 3a 1H NMR spectrum. [file turkjchem-46-4-1055s10.tif]

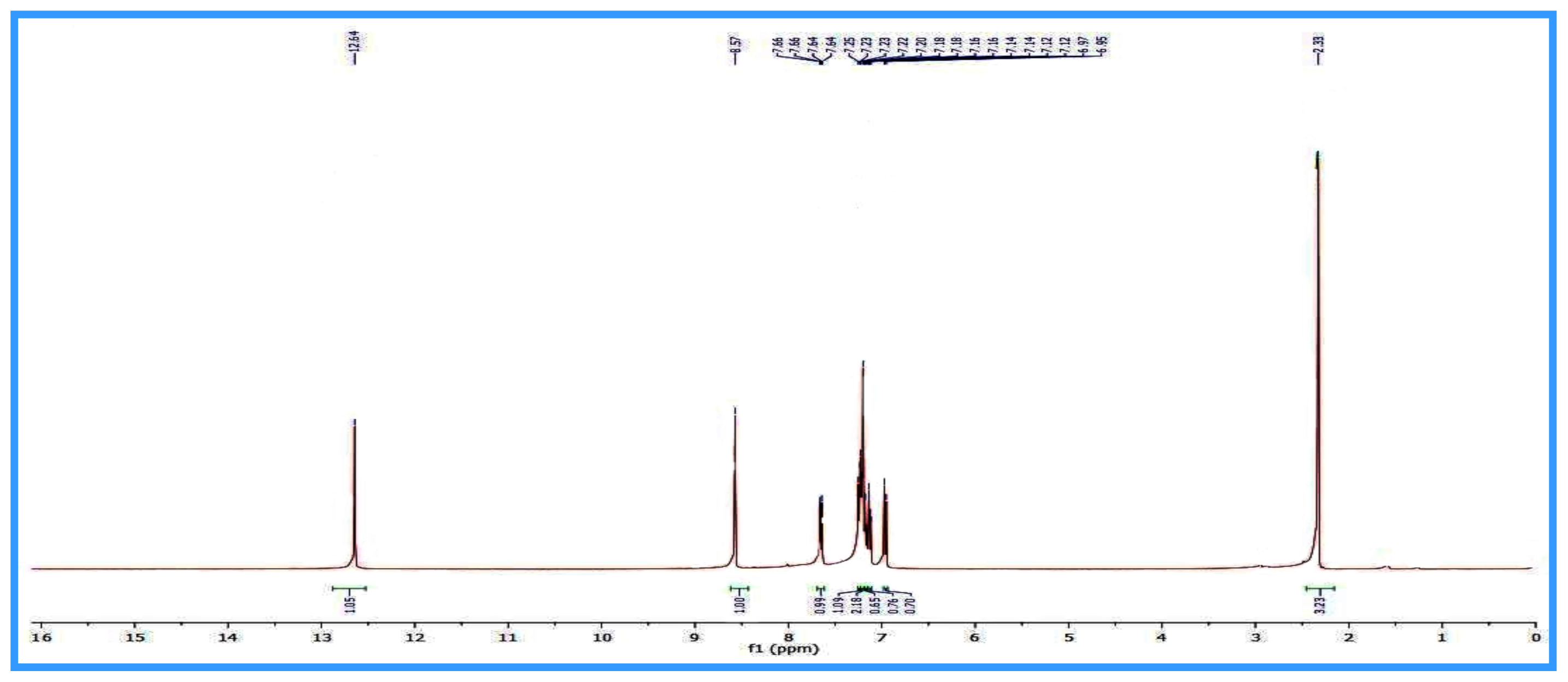

Supplement: Figure S11 — Compound 3b 1H NMR spectrum. [file turkjchem-46-4-1055s11.tif]

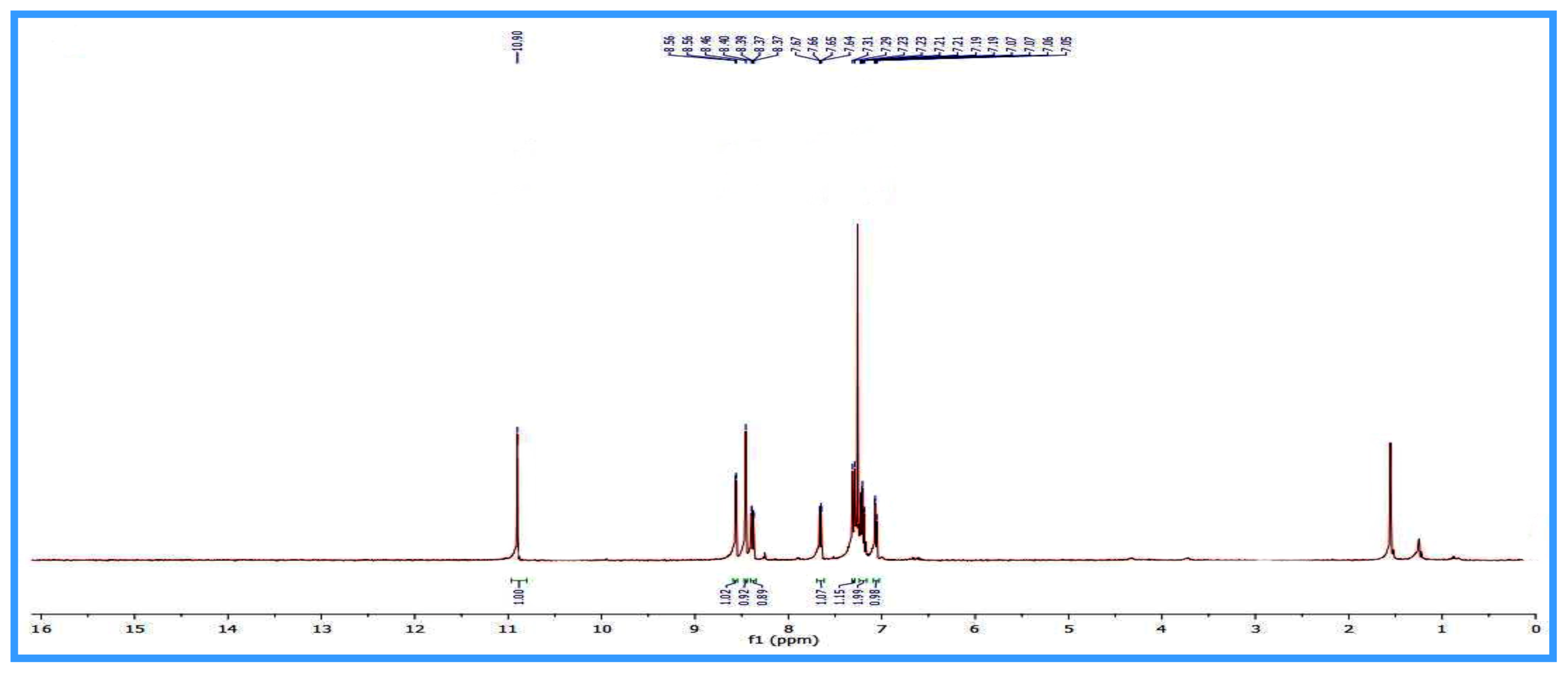

Supplement: Figure S12 — Compound 4c 1H NMR spectrum. [file turkjchem-46-4-1055s12.tif]

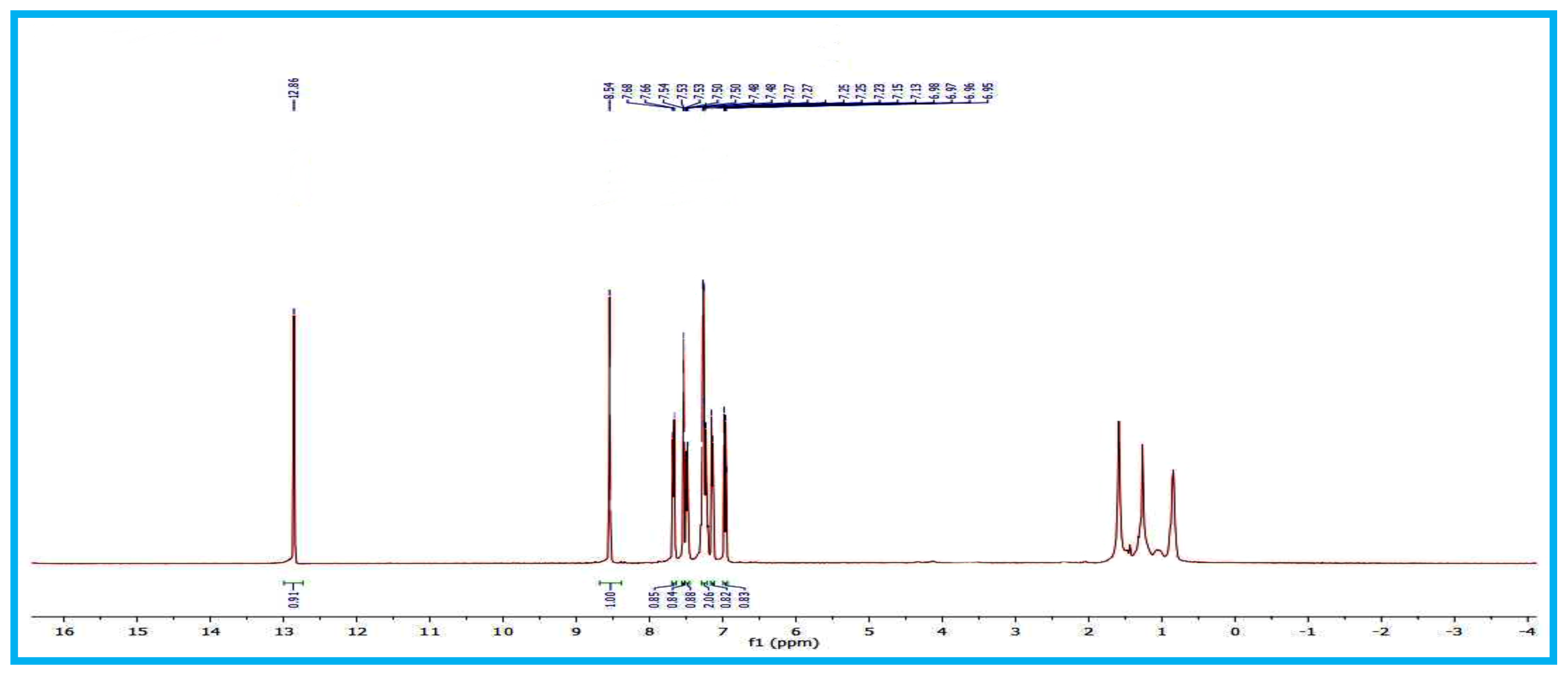

Supplement: Figure S13 — Compound 5 1H NMR spectrum. [file turkjchem-46-4-1055s13.tif]

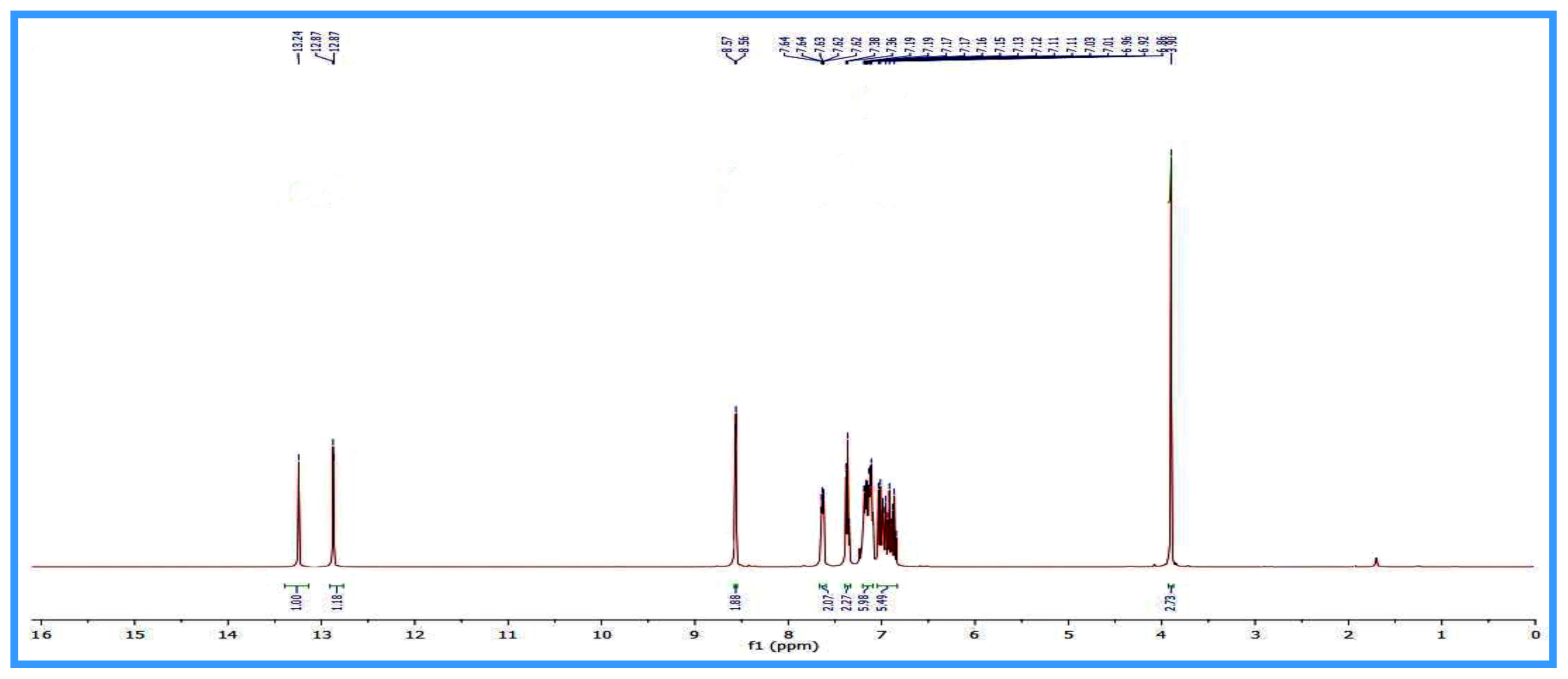

Supplement: Figure S14 — Compound 6 1H NMR spectrum. [file turkjchem-46-4-1055s14.tif]

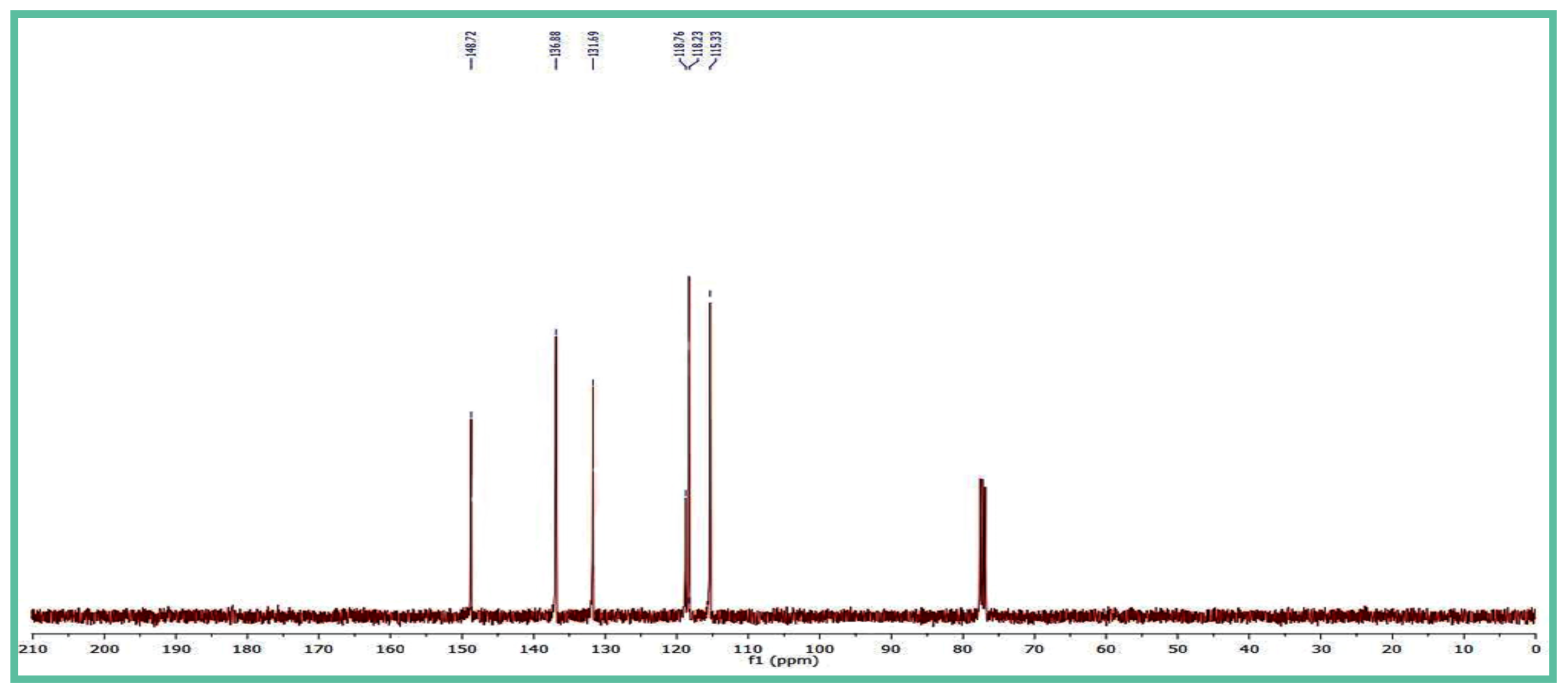

Supplement: Figure S15 — Compound 2 13C NMR spectrum. [file turkjchem-46-4-1055s15.tif]

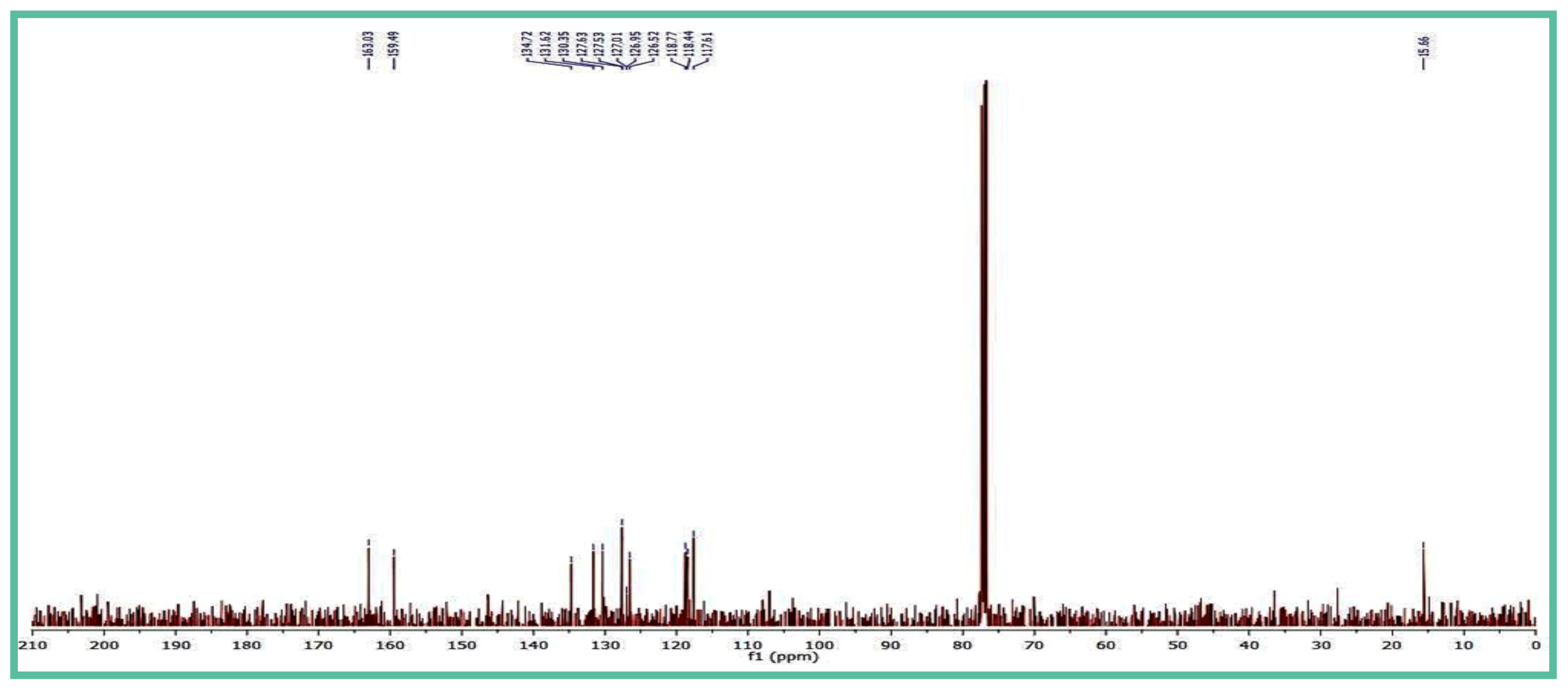

Supplement: Figure S16 — Compound 3a 13C NMR spectrum. [file turkjchem-46-4-1055s16.tif]

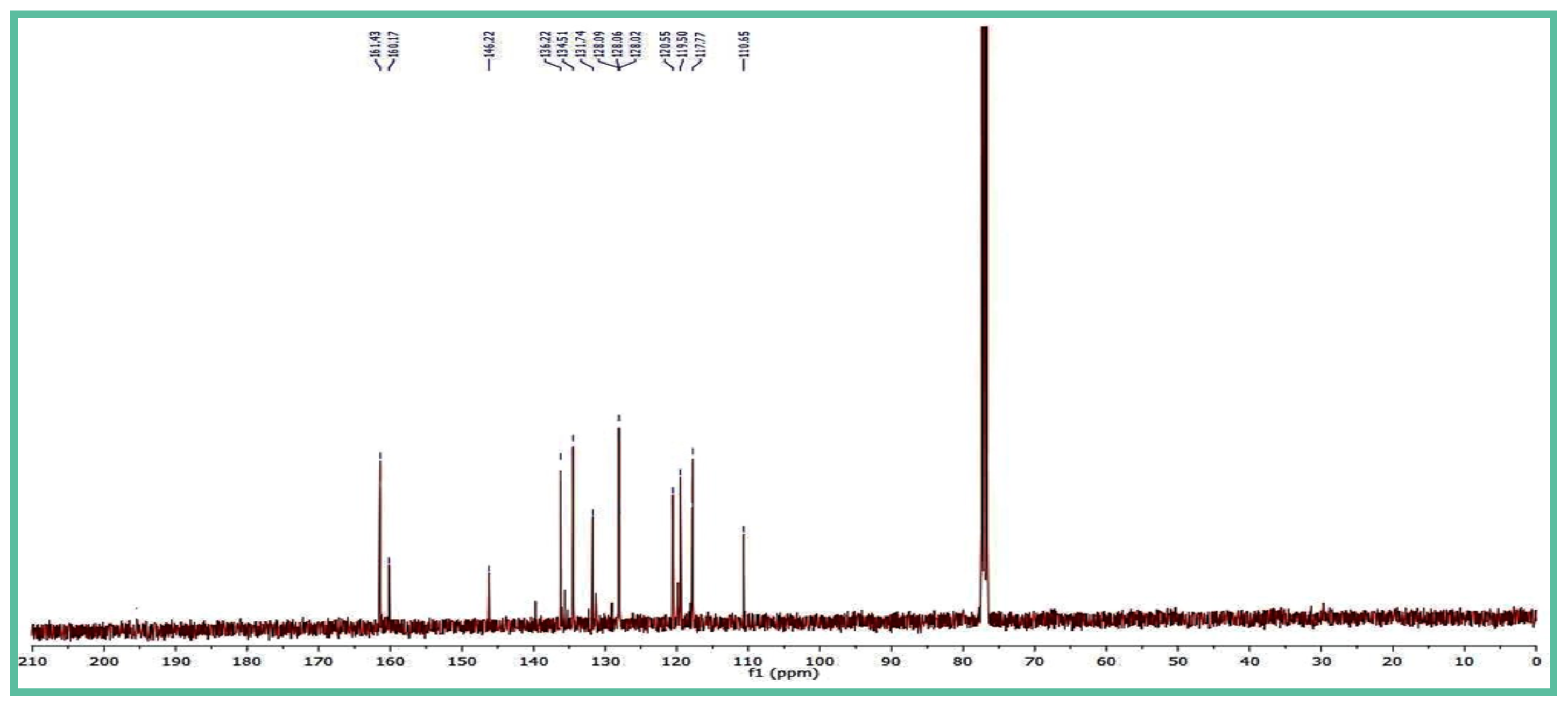

Supplement: Figure S17 — Compound 5 13C NMR spectrum. [file turkjchem-46-4-1055s17.tif]

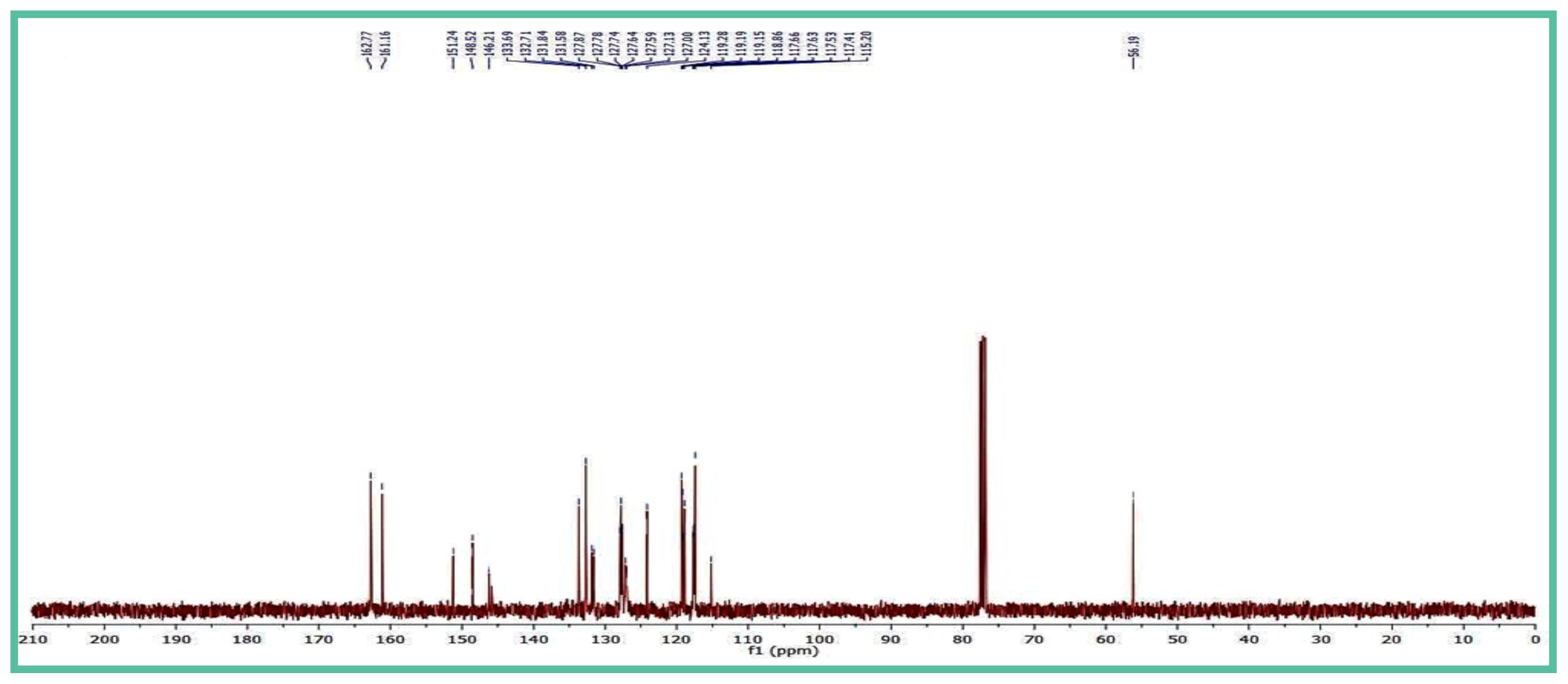

Supplement: Figure S18 — Compound 6 13C NMR spectrum. [file turkjchem-46-4-1055s18.tif]

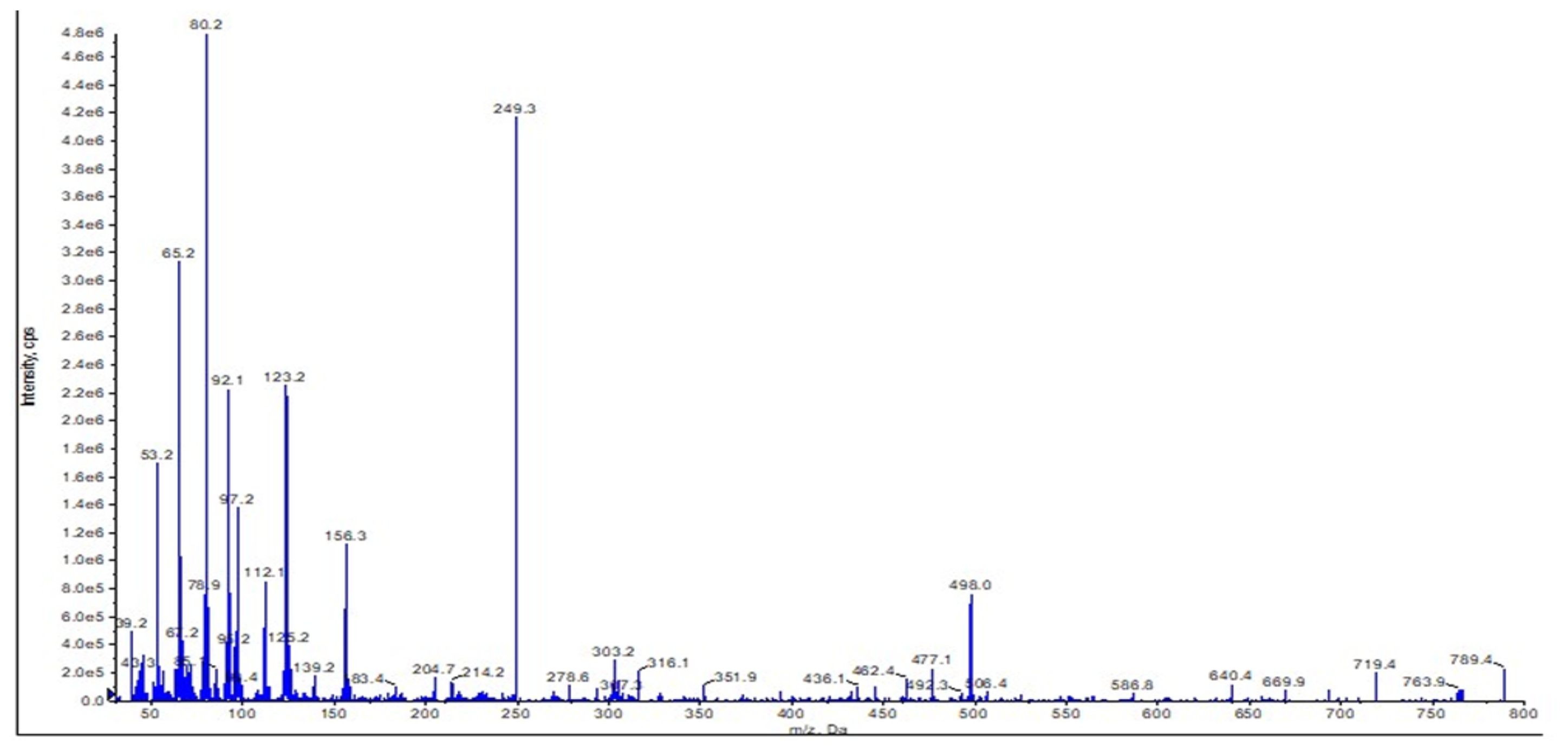

Supplement: Figure S19 — Compound 2 MS spectrum. [file turkjchem-46-4-1055s19.tif]

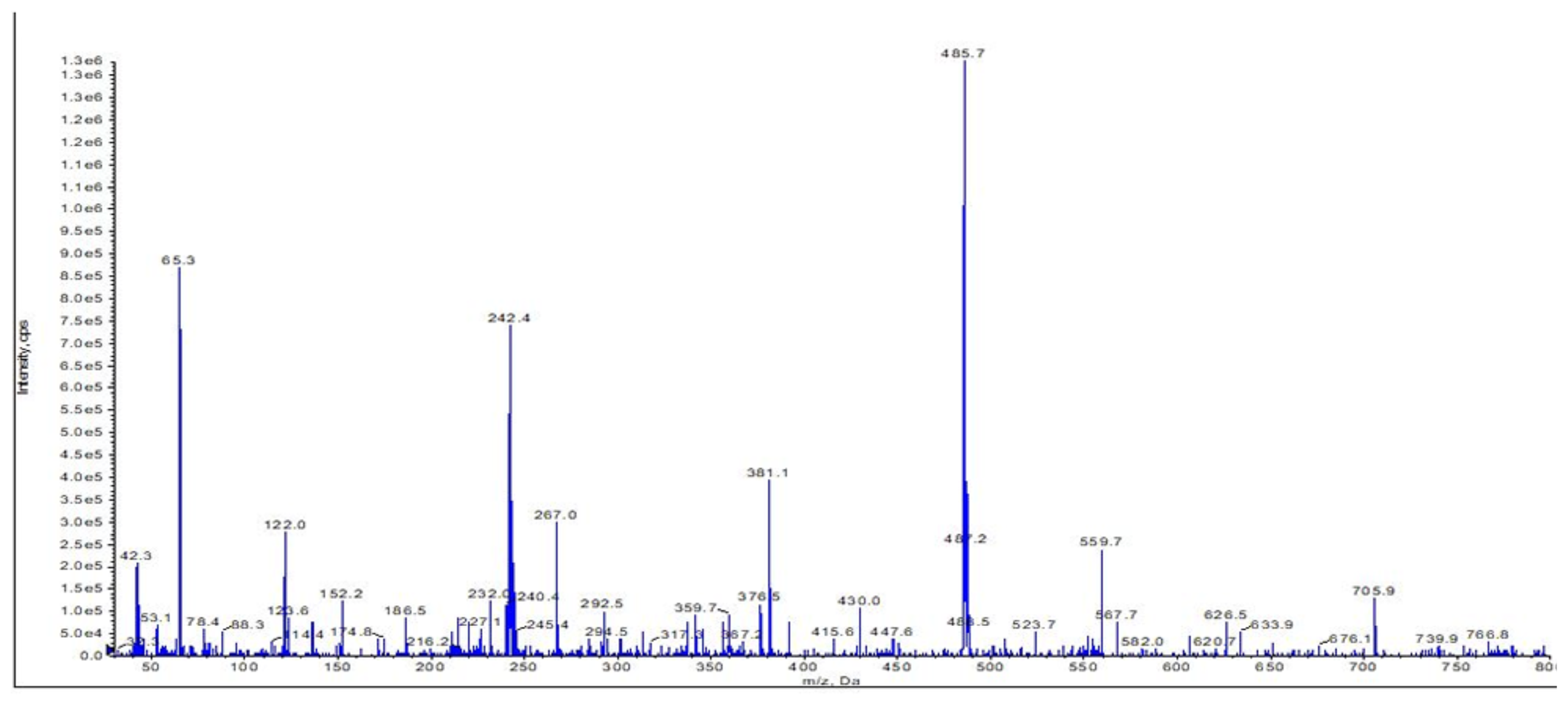

Supplement: Figure S20 — Compound 3b MS spectrum. [file turkjchem-46-4-1055s20.tif]

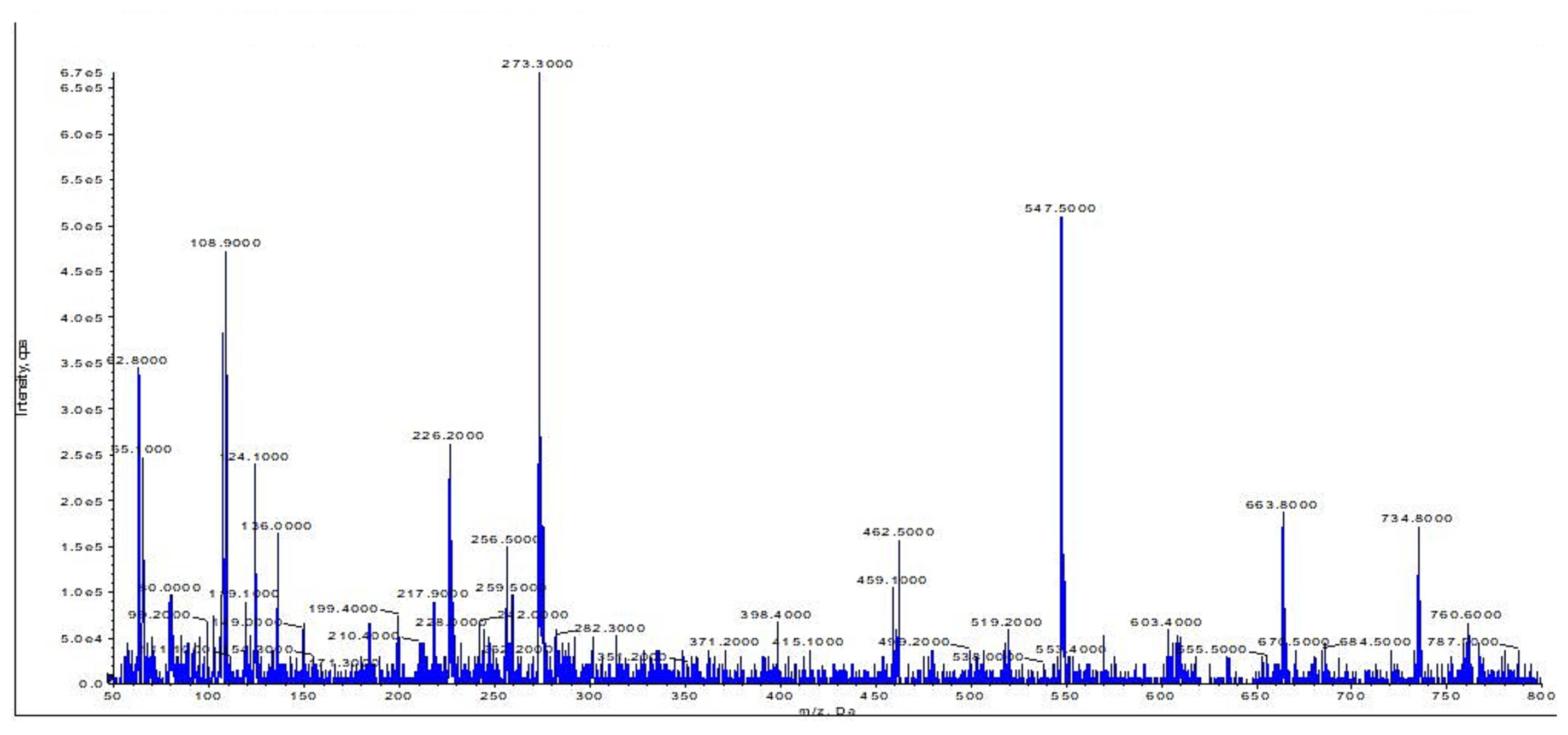

Supplement: Figure S21 — Compound 4a MS spectrum. [file turkjchem-46-4-1055s21.tif]

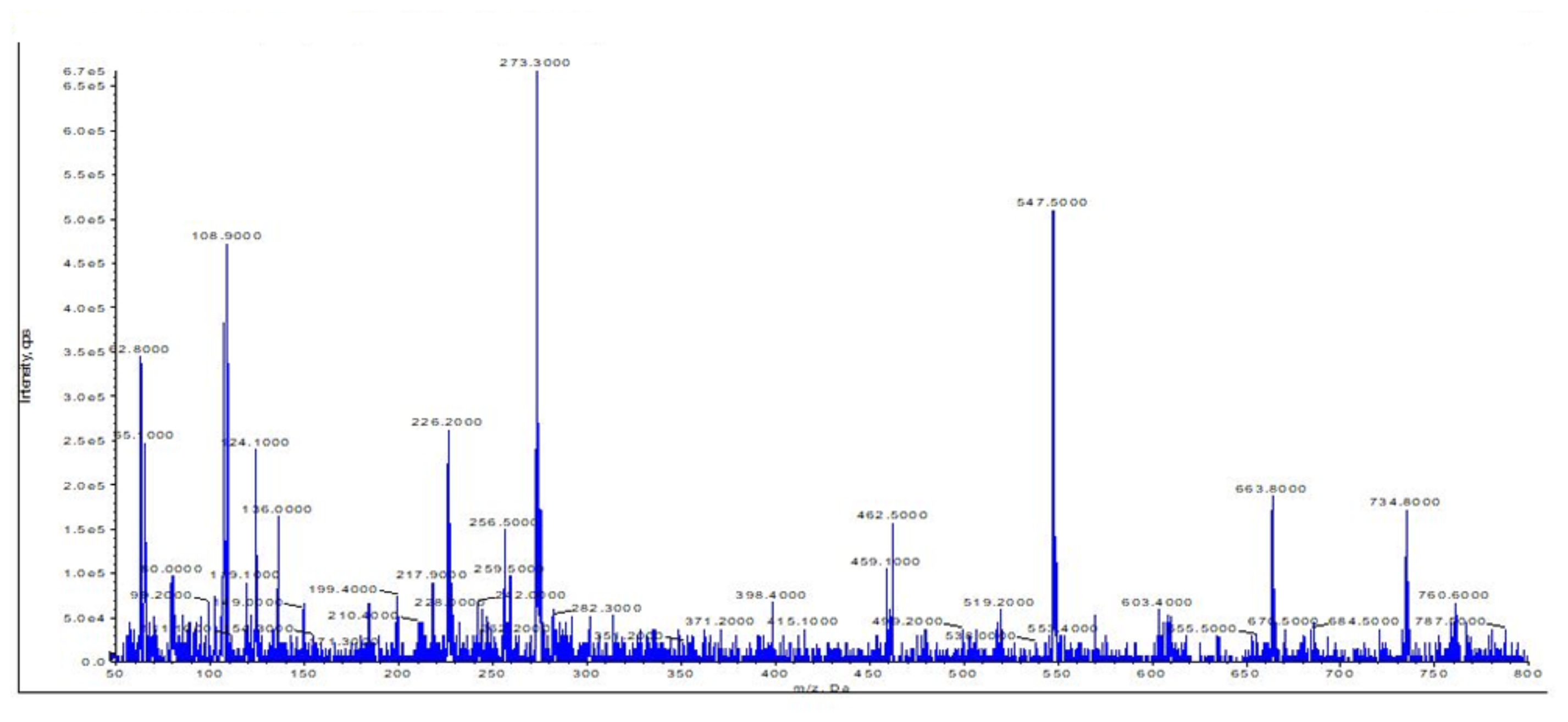

Supplement: Figure S22 — Compound 4b MS spectrum. [file turkjchem-46-4-1055s22.tif]

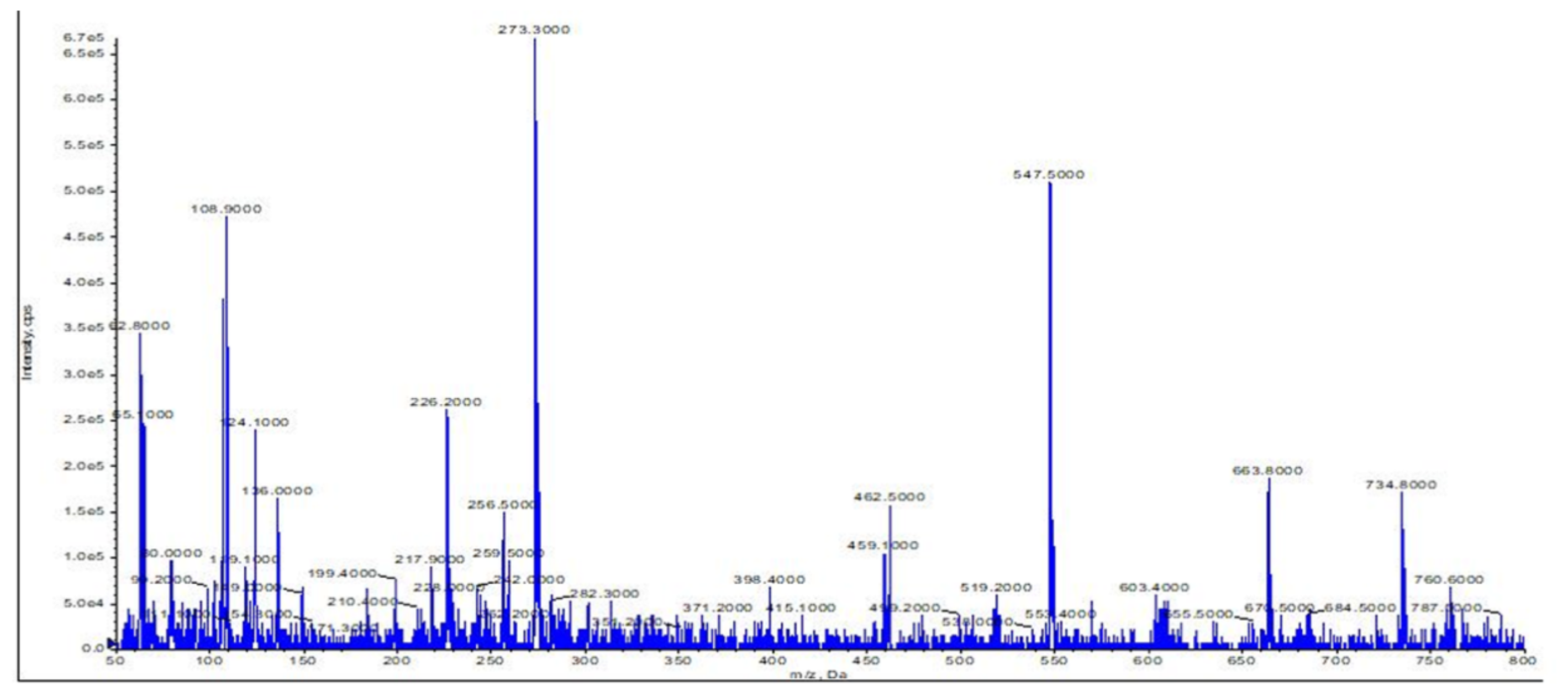

Supplement: Figure S23 — Compound 4c MS spectrum. [file turkjchem-46-4-1055s23.tif]

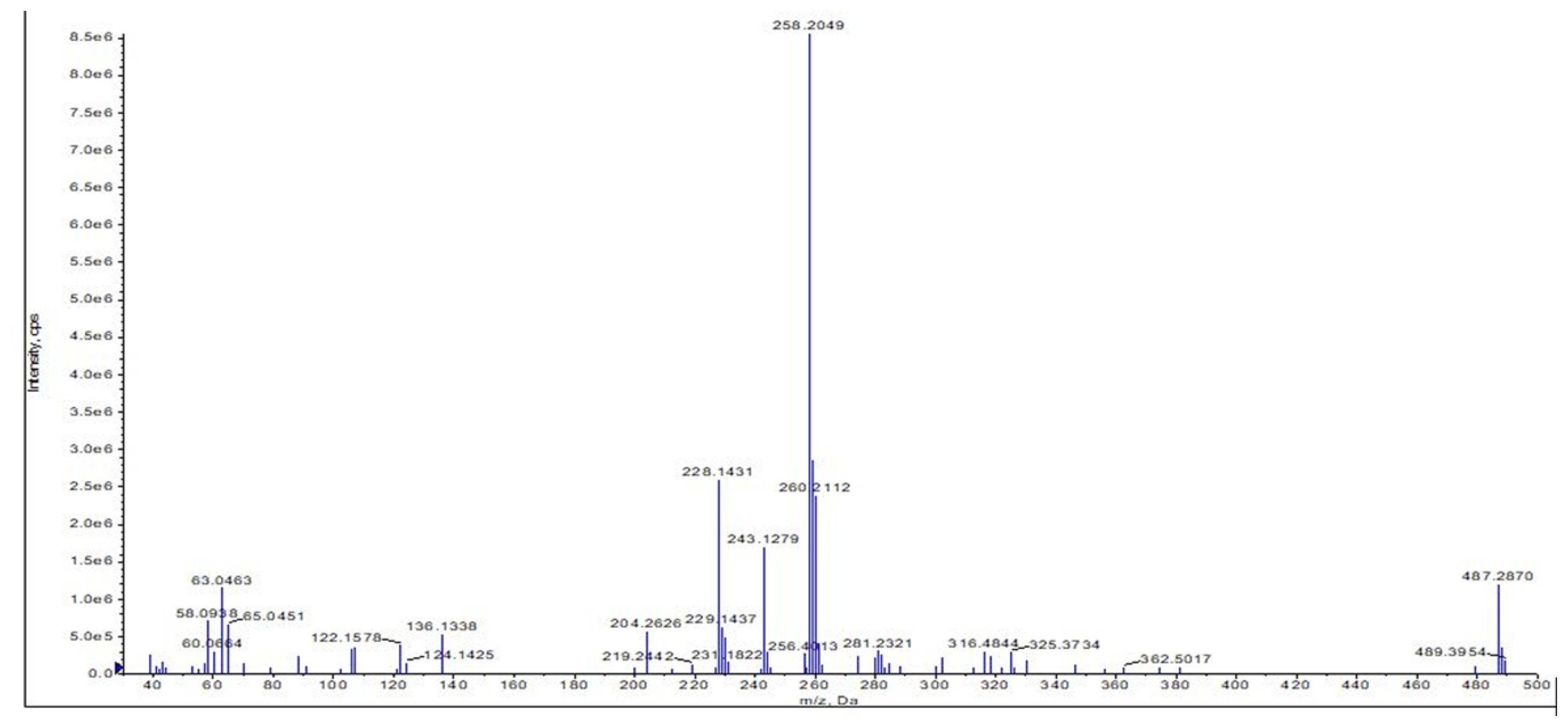

Supplement: Figure S24 — Compound 6 MS spectrum. [file turkjchem-46-4-1055s24.tif]
